# Developmental Toxicity of Tetrabromobisphenol A Bis(allyl ether) in Zebrafish: Redox Disturbance and Osteoclast-Associated Skeletal Responses

**DOI:** 10.3390/antiox15101242

**Published:** 2026-09-26

**Authors:** Lele Fu, Hai Xu, Qing Wei, Yungu Zheng, Ling Cai, Ming Yang

**Affiliations:** 1 School of Environmental and Chemical Engineering, Shanghai University, Shanghai 200444, China; 2 School of Environment and Safety Engineering, Jiangsu University, Zhenjiang 212013, China; 3 Key Laboratory of Marine Ecological Conservation and Restoration, Third Institute of Oceanography, Ministry of Natural Resources, Xiamen 361005, China

**Keywords:** brominated flame-retardant derivative, oxidative imbalance, craniofacial cartilage, skeletal remodeling, osteoclast differentiation, RNA sequencing, non-monotonic concentration response

## Abstract

Tetrabromobisphenol A bis(allyl ether) (TBBPA-BAE) is an emerging aquatic contaminant, yet its developmental toxicity, particularly its stage-dependent skeletal effects, remains poorly understood. Zebrafish embryos and larvae were continuously exposed to nominal concentrations of 0.2, 2, 20, or 200 µg/L TBBPA-BAE (BAE0.2, BAE2, BAE20, and BAE200) through 14 days post-fertilization (dpf), with the lowest concentration approximating the upper end of reported environmental concentrations. Exposure transiently delayed hatching and altered heart rate and locomotion. At 5 dpf, H_2_O_2_ increased at all concentrations, whereas superoxide-scavenging capacity, catalase, and total superoxide dismutase showed distinct concentration-dependent responses, indicating disrupted redox homeostasis rather than uniform antioxidant activation. Selective craniofacial cartilage changes were also observed. At 14 dpf, craniofacial mineralization changed non-monotonically, increasing in BAE2 and BAE20 but decreasing in BAE200. Increased cranial TRAP staining together with osteoclast-related transcriptional changes indicated altered osteoclast-associated remodeling, while RANKL–RANK–OPG-related transcripts showed non-coordinated regulation. BAE20 produced the largest 5 dpf transcriptomic response, with changes involving redox response, extracellular matrix regulation, and osteoclast-related processes. Overall, TBBPA-BAE induced stage-dependent, non-monotonic developmental responses characterized by early redox disturbance and later osteoclast-associated skeletal alterations.

## 1. Introduction

The growing application of structurally modified tetrabromobisphenol A (TBBPA) derivatives has broadened the spectrum of brominated flame retardants entering the environment [1]. Among these derivatives, tetrabromobisphenol A bis(allyl ether) (TBBPA-BAE) is employed as a reactive or additive flame retardant in polymeric materials and differs from the parent compound TBBPA through substitution of both phenolic hydroxyl groups with allyl ether moieties. This structure modification markedly increases the compound’s hydrophobicity, as evidenced by a reported log*K_ow_* of 11.4 compared with 6.5 for TBBPA, and is therefore expected to influence its partitioning behavior, uptake, and biological persistence [1,2]. Although TBBPA-BAE was included in the 2024 European Food Safety Authority reassessment, the available occurrence data and toxicological evidence were insufficient to support a robust risk evaluation [2]. Its environmental relevance, however, is becoming increasingly apparent. Concentrations of up to 0.19 ± 0.01 µg/L in river water and 0.13 ± 0.05 µg/L in wastewater-treatment-plant effluent have been reported [3]. Dietary-exposure experiments further demonstrated its transfer from contaminated prey to zebrafish, followed by metabolic transformation such as ether-bond cleavage and phase-II conjugation, despite the absence of trophic biomagnification [4]. Together, these lines of evidence on environmental occurrence, biological uptake, and inadequate risk characterization underscore the urgent need for compound-specific investigation of TBBPA-BAE.

Evidence from TBBPA and other derivatives indicates that developmental toxicity can vary substantially even among closely related structures. In zebrafish, the parent compound has been linked to impaired cerebral and ocular vascular development, altered mitochondrial function and elevated reactive oxygen species during embryogenesis [5]. Exposure beginning during early cleavage also slowed developmental progression and interfered with histone H3 lysine 27 acetylation and zygotic genome activation, suggesting that the maternal-to-zygotic transition is a sensitive developmental window [6]. However, these findings cannot be transferred directly to every TBBPA derivative. Comparative experiments showed that TBBPA caused clear developmental toxicity under conditions in which TBBPA-bis(2,3-dibromopropyl ether) produced relatively limited morphological effects, despite both compounds affecting cardiac function [7]. TBBPA-bis(2-hydroxyethyl ether) has also been associated with altered locomotion, oxidative imbalance and impaired craniofacial and neural development [8,9]. Reported transcriptional changes involved biological processes related to neural patterning, synaptic organization, neuronal differentiation, glial development and axonal growth, represented by genes such as *shha*, *syn2a*, *elavl3*, *gfap* and *gap43* [9]. Collectively, these studies show that modification of the TBBPA structure can change both toxic potency and the biological systems affected. Direct testing of TBBPA-BAE is therefore necessary rather than inferring its effects from the parent compound or another derivative.

Zebrafish provide a useful framework for linking early functional and biochemical responses with structural changes that emerge later in development. Research on TBBPA-related compounds has largely concentrated on mortality, neurodevelopment, vascular formation, epigenetic regulation and behavior, whereas skeletal development and remodeling have received comparatively little attention. Craniofacial cartilage is particularly responsive to developmental toxicants, including halogenated bisphenols, and can reveal selective structural changes before overt malformation becomes apparent [10,11]. Assessment of later mineralization and osteoclast-associated activity adds information on skeletal maturation and remodeling that cannot be obtained from cartilage staining alone [12]. Altered redox homeostasis may also be involved in skeletal remodeling. As both a marker of oxidative imbalance and a signaling mediator of osteoclast differentiation, hydrogen peroxide drives osteoclastogenesis, whereas antioxidant systems mitigate oxidative stress to limit bone resorption [13]. Notably, osteoclast maturation depends not solely on redox status but also on balanced activity within the RANKL-RANK-OPG axis, and cannot be assessed using any isolated transcriptional or staining readout [14,15]. Concentration–response relationships may further be complicated by adaptive feedback, differences in toxicokinetics and concentration-dependent engagement of molecular targets, producing responses that peak at intermediate concentrations or change direction at higher exposures [16,17]. These considerations support an assessment that integrates multiple skeletal, redox and molecular endpoints across both concentration and developmental stage.

The developmental effects of TBBPA-BAE, particularly those involving skeletal development and remodeling, remain poorly defined. It is also unclear how early physiological, redox and transcriptomic responses relate to skeletal phenotypes observed at later developmental stages. We therefore hypothesized that developmental exposure to TBBPA-BAE would elicit concentration- and stage-dependent biological responses in zebrafish, with potential effects on early development and skeletal homeostasis. To test this hypothesis, zebrafish were continuously exposed to 0.2, 2, 20 or 200 µg/L TBBPA-BAE from 5 h post-fertilization (hpf) to 14 days post-fertilization (dpf). Early developmental, cardiovascular, locomotor, redox, cartilage and transcriptomic endpoints were assessed at or before 5 dpf, followed by mineralization, cranial tartrate-resistant acid phosphatase staining and targeted osteoclast-related gene expression at 14 dpf. The transcriptomic analyses were used to characterize molecular responses and prioritize candidate processes for further investigation.

## 2. Materials and Methods

### 2.1. Chemicals and Reagents

Tetrabromobisphenol A bis(allyl ether) (TBBPA-BAE; CAS 25327-89-3; >99% purity) and dimethyl sulfoxide (DMSO; CAS 67-68-5; ≥99.9% purity) were obtained from Sangon Biotech (Shanghai, China). Both reagents were of analytical grade.

### 2.2. Zebrafish Husbandry and Embryo Collection

Wild-type TU zebrafish from the China Zebrafish Resource Center (Wuhan, China) were maintained at 28 ± 0.5 °C under a 14 h light/10 h dark cycle. Fish were fed *Artemia* twice daily. Six-month-old fish were paired at a 1:2 male-to-female ratio, and viable fertilized embryos were collected 30–40 min after spawning. All zebrafish procedures were approved by the Ethics Committee of Shanghai University (Approval No. ECSHU 2023-081; approved on 13 October 2023).

### 2.3. Embryo Exposure Design

A 2 mg/mL TBBPA-BAE stock in DMSO was serially diluted in E3 medium to nominal concentrations of 0.2, 2, 20 and 200 µg/L (BAE0.2, BAE2, BAE20 and BAE200). All groups, including the vehicle control, contained 0.01% (*v*/*v*) DMSO. BAE0.2 was selected to approximate the upper range of reported aquatic concentrations (0.19 ± 0.01 μg/L) [3]; higher concentrations formed a 10-fold series for sub-lethal hazard characterization. The highest concentration, 0.2 mg/L, remained substantially below the previously reported 96 h maximal non-lethal concentration (120.2 mg/L) and LC_50_ (184.0 mg/L) for TBBPA-BAE [8].

At 5 hpf, viable embryos were randomly assigned to control and exposure groups and maintained at 28 °C. Fresh exposure solutions were prepared and completely renewed every 24 h. From 5 dpf, larvae were fed freshly hatched *Artemia* twice daily; residual food and debris were removed during renewal. Water-quality parameters under the exposure conditions are provided in Supplementary Table S1. At 5 dpf, larvae were collected and snap-frozen in liquid nitrogen, and then stored at −80 °C for later transcriptome analyses.

Each endpoint was evaluated in a separate cohort over three independent experimental runs, with each run initiated from a separate spawning event. Within each run, all treatment groups were derived from the same spawning batch and processed in parallel. Individual-exposure assays, including hatching, heart rate, locomotor behavior, cartilage staining, mineralization and TRAP staining, used one embryo or larva in 5 mL of exposure medium per well in plastic 12-well plates. Group-exposure assays, including redox measurements, RT-qPCR and RNA-seq, used 20 larvae in 10 mL of medium per well in plastic six-well plates. Replication, plate allocation and sampling procedures for each endpoint are summarized in Supplementary Table S2.

### 2.4. Heart Rate

At 96 hpf, 12 larvae per treatment, each maintained in a separate exposure well from the beginning of exposure, were briefly transferred to fresh E3 medium containing 0.287 g/L NaCl, 0.0127 g/L KCl, 0.0483 g/L CaCl_2_·2H_2_O and 0.0817 g/L MgSO_4_·7H_2_O. Larvae were immobilized laterally in 6% methylcellulose, and cardiac activity was video-recorded using the ZebraCube system v5.13.0.30 (ViewPoint, Lyon, France). Heart rate was determined by counting the number of heartbeats over 1 min.

### 2.5. Locomotor Behavior

At 120 hpf, 12 larvae per treatment, each originating from a separate exposure well containing one larva, were transferred individually into 48-well plates containing 1 mL of E3 medium per well. After 10 min of acclimation under white light, basal locomotor activity was recorded for 10 min. Swimming trajectories and mean swimming speed were quantified using ZebraCube ZEB6118-1 software-equipped observation cube for adult zebrafish behavior (ViewPoint, Lyon, France). Mean swimming speed was normalized to the control-group mean. Movement was classified according to instantaneous swimming speed as slow/immobile (<2 mm s^−1^), intermediate (2–10 mm s^−1^) or fast (>10 mm s^−1^). For each larva, the proportion of time spent in each movement state was calculated over the 600 s recording period.

### 2.6. Redox-Related Biochemical Assays

At 5 dpf, 20 larvae from each exposure well were pooled and homogenized in phosphate-buffered saline, yielding six well-derived biological replicates per treatment (2 wells/run × 3 runs). Commercial kits (Nanjing Jiancheng Bioengineering Institute, Nanjing, China) were used to quantify H_2_O_2_, superoxide-scavenging capacity, hydroxyl-radical-related activity, catalase (CAT) and total superoxide dismutase (T-SOD). Assay principles, catalog numbers and detection wavelengths are listed in Supplementary Table S3.

### 2.7. Cartilage, Bone and TRAP Staining

Alcian blue, Alizarin red S and tartrate-resistant acid phosphatase (TRAP) staining were used to assess craniofacial cartilage, mineralization and cranial osteoclast-associated staining, respectively. Larvae were fixed in 4% paraformaldehyde and imaged ventrally under identical settings. Cartilage was assessed at 5 dpf after Alcian blue staining for 17 h at room temperature (24–30 °C), followed by acidified-ethanol destaining and KOH/glycerol clearing. Cartilage-positive area and prespecified craniofacial dimensions were quantified. Mineralization was assessed at 14 dpf using 0.05% Alizarin red S for 30 min at room temperature (24–30 °C) (*n* = 12 larvae per treatment for each endpoint). Mineralized area and integrated optical density (IOD) were quantified. For size normalization, mineralized area was divided by the corresponding craniofacial area of each larva, and the resulting ratio was expressed relative to the control-group mean. At 14 dpf, TRAP staining used a commercial chromogenic kit (Suzhou Murui Biotechnology Co., Ltd., Suzhou, China) at 37 °C for 60 min, with simultaneous initiation and termination across groups (*n* = 6 larvae per treatment). TRAP-positive area was quantified within the cranial region. Images were quantified using ImageJ v1.54s [18] with a 200 µm calibration and consistent thresholds within each assay. All images, representative-image selections and quantitative measurements were independently reviewed by a second author blinded to treatment. Unless otherwise specified, non-angular measurements were expressed relative to the concurrent control mean. Additional craniofacial morphometric endpoints are provided in Supplementary Figure S1.

### 2.8. Targeted RT-qPCR Analysis

At 14 dpf, three pooled biological replicates were collected per treatment, one from each independent run. Each replicate consisted of 20 larvae pooled from four exposure wells, with five larvae sampled from each well. Each exposure well contained 20 larvae during the exposure period. RNA was extracted using RNAiso Plus (Takara Bio Inc., Kusatsu, Japan), RNA concentration and purity were assessed using a NanoDrop 2000 (Thermo Fisher Scientific, Waltham, MA, USA). cDNA was synthesized using PrimeScript RT Master Mix (Takara Bio Inc.). RT-qPCR was performed in 20 µL reactions on a CFX96 Real-Time PCR Detection system (Bio-Rad Laboratories, Hercules, CA, USA). The cycling program consisted of 95 °C for 30 s, followed by 40 cycles of 95 °C for 5 s and 60 °C for 30 s.

We analyzed the expression of selected target genes including *acp5a* (acid phosphatase 5a, tartrate resistant), *fosab* (Fos proto-oncogene, AP-1 transcription factor subunit b), *ctsk* (cathepsin K), *nfatc1* (nuclear factor of activated T cells 1) and *ocstamp* (osteoclast stimulatory transmembrane protein), as well as genes closely related to the RANKL–RANK–OPG axis, including *tnfsf11* (tumor necrosis factor ligand superfamily member 11; RANKL), *tnfrsf11a* (tumor necrosis factor receptor superfamily member 11a; RANK) and *tnfrsf11b* (tumor necrosis factor receptor superfamily member 11b; OPG). *actb* (actin beta) was used as the reference gene. Primer sequences are provided in Supplementary Table S4. Relative expression was calculated using the 2^−ΔΔCt^ method [19], with the control mean set to 1. The mRNA-based RANKL/OPG ratio was calculated for each biological replicate as the relative expression of *tnfsf11* divided by that of *tnfrsf11b*, and group values are presented as mean ± s.e.m. of the three replicate ratios.

Reactions were run in technical triplicate, with melting-curve analysis and no-template controls. Primer efficiencies, reaction-exclusion criteria, plate allocation and reference-gene Cq data are provided in Supplementary Tables S4 and S5; standard curves and melting profiles are shown in Supplementary Figures S2 and S3.

### 2.9. RNA Sequencing, Pathway Enrichment and Network Analyses

For RNA sequencing (RNA-seq), 100 zebrafish larvae were pooled as one biological sample, with three independent biological replicates per treatment. Each RNA-seq biological replicate comprised 100 larvae pooled from ten independent exposure wells, with ten larvae collected from each well. Total RNA was extracted using TRIzol (Invitrogen, Carlsbad, CA, USA) and purified using the RNeasy Mini Kit with on-column DNase treatment (Qiagen, Hilden, Germany). Each replicate was processed and sequenced separately.

Poly(A)-enriched libraries were prepared and sequenced by Majorbio Bio-pharm Technology Co., Ltd. (Shanghai, China) on an Illumina NovaSeq platform using paired-end 151 bp reads. Reads were quality-controlled using SeqPrep and fastp v0.23.4 and aligned to the *Danio rerio* reference genome using HISAT2 v2.2.1. Library preparation and sample-level sequencing and mapping metrics are detailed in Supplementary Table S6. Raw reads are available under BioProject PRJNA1512095 (SRR40145874–SRR40145888).

Gene expression was quantified using RSEM [20]. Gene-level counts were analyzed using DESeq2 v1.42.0 [21], with formal DEGs defined by Benjamini–Hochberg-adjusted *p* < 0.05 and absolute fold change ≥1.5 (|log_2_FC| ≥ 0.585). TPM values were used for PCA, selected-gene expression displays and WGCNA.

For exploratory IPA (QIAGEN; client release 01-23-01), genes with nominal DESeq2 *p* < 0.05 and absolute fold change ≥1.5 were used to retain sensitivity to coordinated pathway-level changes. IPA input identifier composition and recurrent-pathway selection are detailed in Supplementary Table S7. IPA results were treated as hypothesis-generating molecular signatures. Complementary KEGG GSEA used the full gene list ranked by Signal2Noise for each exposure-versus-control comparison, with gene-set sizes of 15–500 and adjusted *p* < 0.05 (Supplementary Figure S4).

Selected-gene TPM values were log_2_(TPM + 1)-transformed and standardized within each gene across all 15 samples. Gene-set membership and classification are provided in Supplementary Table S8; these displays were descriptive.

Exploratory WGCNA [22] used the TPM matrix from all 15 samples on the Majorbio Cloud Platform (signed network, β = 9, minimum module size = 15, mergeCutHeight = 0.05). Genes from modules positively associated with BAE20 and with module membership ≥0.30 were intersected with BAE20 DEGs and analyzed using STRING for *Danio rerio* [23]. This was same-dataset candidate prioritization, not independent validation. Fixed-module leave-one-sample-out sensitivity analysis recalculated module eigengenes and their Spearman correlations with each treatment group after omitting one sample at a time (Supplementary Table S9).

### 2.10. Statistical Analysis

Quantitative data are presented as mean ± s.e.m. Statistical analyses were performed according to endpoint type and data structure, with detailed procedures, multiple-comparison corrections and hypothesis families provided in Supplementary Table S10. RT-qPCR analyses were based on ΔCt values, and WGCNA *p* values were treated as nominal. Analyses and visualization were performed using Python 3.14.4 and GraphPad Prism 10.4.

## 3. Results

### 3.1. TBBPA-BAE Exposure Altered Hatching, Heart Rate and Swimming Behavior

Hatching rates differed significantly among groups at 54 hpf (*p* < 0.001; Figure 1A). All exposure groups showed lower hatching rates than the control at this time point, with the largest reductions observed in the BAE20 and BAE200 groups. By 72 hpf, hatching rates had largely recovered and remained close to the control level at 96 hpf, indicating a temporary delay rather than a persistent failure of hatching. Heart rate also differed among groups (*p* = 0.008; Figure 1B). A significant increase was observed in the BAE0.2 group, whereas the BAE2, BAE20 and BAE200 groups did not differ significantly from the control.

The representative swimming tracks showed dense fast-movement trajectories in the control, BAE0.2 and BAE2 groups, whereas slower and less extensive movement was more evident in the BAE20 and BAE200 groups (Figure 1C). Consistent with these observations, relative swimming speed differed significantly among groups (*p* < 0.001; Figure 1D). Swimming speed was reduced in the BAE20 group and decreased further in the BAE200 group, while no significant change was detected at 0.2 or 2 µg/L.

Swimming-state composition differed significantly from the control in all exposure groups (Figure 1E). Descriptively, the mean proportion of slow/immobile movement increased, whereas the proportion of fast movement decreased, with the largest shifts observed in the BAE20 and BAE200 groups. Intermediate-speed movement varied among groups without a consistent concentration-dependent pattern. Overall, TBBPA-BAE shifted swimming behavior toward slower or inactive states, particularly at the two highest concentrations.

### 3.2. TBBPA-BAE Exposure Disrupted Redox Homeostasis During Early Development

TBBPA-BAE exposure significantly altered all five redox-related parameters, but the pattern of change differed among them (Figure 2). H_2_O_2_ levels increased at all tested concentrations, with the highest mean values observed in the BAE20 and BAE200 groups (Figure 2A). Superoxide-scavenging capacity also increased, with the greatest response observed in the BAE20 group (Figure 2B). The remaining redox-related measures showed different concentration-dependent patterns. Hydroxyl-radical-related activity increased mainly in BAE2, where CAT activity also increased (Figure 2C,D). CAT activity decreased below the control level in BAE200. T-SOD activity increased in BAE2 and BAE20 but returned toward the control level in BAE200 (Figure 2E). Together, these different response patterns indicate disrupted redox homeostasis rather than a uniform antioxidant response.

### 3.3. TBBPA-BAE Exposure Altered Craniofacial Cartilage and Bone Mineralization

TBBPA-BAE exposure produced selective changes in craniofacial cartilage development (Figure 3A–C). Cartilage-positive area differed significantly among groups (*p* < 0.001; Figure 3B). It was increased in the BAE20 and BAE200 groups, with the larger increase observed in BAE20. Craniofacial cartilage height was also affected (*p* = 0.016; Figure 3C), with significant increases in the BAE2 and BAE20 groups. These results indicate that TBBPA-BAE altered craniofacial cartilage morphology, particularly at intermediate exposure concentrations.

The CH–PQ, CH–CH, PQ–Meckel’s and Meckel’s–Meckel’s angles showed no overall differences. Head length, eye diameter and eye depth were also unchanged (Supplementary Figure S1). Although interocular distance differed in the omnibus test, no control comparison remained significant after Holm correction. Overall, these findings indicate localized changes in craniofacial cartilage rather than an overt craniofacial malformation.

Craniofacial bone mineralization differed significantly among groups at 14 dpf (Figure 3D–G). The unnormalized mineralized area increased in BAE2 and BAE20 but decreased in BAE200 relative to the control (*p* < 0.001; Figure 3E). Control-normalized Alizarin red IOD showed a similar pattern (*p* < 0.001; Figure 3F). Craniofacial area did not differ significantly among groups (*p* = 0.4144; Supplementary Figure S5A), and no significant association was detected between craniofacial area and unnormalized mineralized area across individuals (Pearson r = −0.043, permutation *p* = 0.7434; Supplementary Figure S5B). After mineralized area was normalized to the corresponding craniofacial area, a significant overall group effect remained (*p* < 0.001; Figure 3G). The increases in BAE2 and BAE20 remained significant, whereas BAE200 no longer differed significantly from the control.

### 3.4. TBBPA-BAE Exposure Altered Osteoclast-Associated Responses at 14 dpf

Cranial TRAP-positive area and *acp5a* expression showed overlapping responses across the low-to-intermediate exposure groups (Figure 4A,B). TRAP-positive area increased in BAE0.2, BAE2 and BAE20 and reached its highest value in BAE2. *acp5a* expression was also elevated in these three groups. In BAE200, both endpoints returned toward or below the corresponding control levels. Together, the staining and mRNA expression results indicated concentration-dependent changes in osteoclast-associated endpoints.

The remaining osteoclast-associated genes showed different response patterns (Figure 4C). Neither *fosab* nor *ctsk* differed significantly from the control, whereas *nfatc1* and *ocstamp* were selectively increased in BAE20. Thus, the measured genes did not show coordinated induction across the osteoclast-associated panel.

To place these changes in the context of the RANKL–RANK–OPG system, the mRNA expression of *tnfsf11*, *tnfrsf11a* and *tnfrsf11b*, which encode RANKL, RANK and the decoy receptor OPG, respectively, was examined. The individual transcript levels and the derived RANKL/OPG ratio were treated as expression-based readouts rather than direct measures of signaling activity. *tnfsf11* expression increased in several exposure groups, whereas *tnfrsf11a* was elevated primarily in BAE20 (Figure 4D). *tnfrsf11b* also showed its highest mean expression in BAE20. The mRNA-based RANKL/OPG ratio was higher than the control value in BAE0.2 and BAE200 but lower in BAE2 and BAE20 (Supplementary Table S11). In BAE20, the increases in the RANKL- and RANK-encoding transcripts were accompanied by a larger increase in the OPG-encoding transcript. Overall, the three genes exhibited a mixed expression pattern rather than coordinated induction of the RANKL–RANK–OPG system.

### 3.5. RNA-seq Revealed the Largest Number of DEGs in BAE20 at 5 dpf

At 5 dpf, BAE20 samples separated from the control samples along PC1 (34.9% of the variance; Figure 5A) and had the largest number of formal DEGs: 308 upregulated and 348 downregulated genes (656 total; Figure 5B). The corresponding upregulated/downregulated counts were 77/6 in BAE0.2, 47/15 in BAE2 and 3/5 in BAE200.

Exploratory IPA, based on broader concentration-specific gene lists defined by nominal DESeq2 *p* < 0.05 and an absolute fold change of at least 1.5, identified recurrent pathway-level responses across the exposure groups. The 15 highest-ranked recurrent pathways encompassed inflammatory and cytokine signaling, leukocyte recruitment, wound- and matrix-related processes, lipid and nuclear-receptor regulation, and activation of the pre-replicative complex (Figure 5C). Disease-labeled pathways represent overlapping molecular signatures rather than evidence of the corresponding diseases in zebrafish. Complementary ranked-set KEGG GSEA using the full gene list identified significant concentration-specific pathway enrichment, including several DNA replication and repair pathways (Supplementary Figure S4).

Selected gene sets were examined in relation to the measured phenotypes and pathway-level responses (Figure 5D,E). Redox-response-related and cartilage/bone-matrix gene sets showed concentration-dependent expression changes that were considered alongside the corresponding biochemical and structural findings at 5 dpf. Inflammation/AP-1-related expression shifts identified additional early stress-related molecular changes, although inflammatory phenotypes were not measured directly.

Phenotype-guided examination of cartilage/bone-matrix, osteoclast differentiation/function and *tnfsf11*/RANKL-related genes identified concentration-specific expression patterns relevant to the later skeletal assays, with the clearest shifts in BAE20. These patterns highlight early molecular changes that may be relevant to the skeletal responses observed at 14 dpf and warrant validation at intermediate developmental stages. The DNA-replication/cell-cycle category was retained as a separate exploratory signal because replication-related processes were also evident in the global pathway analysis.

### 3.6. Exploratory WGCNA Highlighted a BAE20-Associated Replication and Cell-Cycle Candidate Network

The network analysis was conducted separately from the targeted early-response comparison. Across 15 samples, exploratory WGCNA identified MEturquoise and MEblue as the modules most positively correlated with BAE20 (Spearman ρ = 0.694 and 0.656; Figure 6A). The two modules contained 4015 and 1813 genes, respectively. Module–trait *p* values were nominal, and the associations were used to prioritize candidates. Leave-one-sample-out analysis showed that BAE20 remained the treatment most strongly positively associated with both MEturquoise and MEblue across all 15 iterations. The corresponding Spearman correlations ranged from 0.608 to 0.713 for MEturquoise and from 0.557 to 0.713 for MEblue (Supplementary Table S9).

Intersecting the two modules with BAE20 DEGs yielded a STRING network organized around DNA replication, mitotic progression or checkpoint control, and chromosome condensation or SMC complexes (Figure 6B). The highest-degree candidates were *kif11*, *cdc20*, *nuf2*, *smc2*, *mcm4*, *smc4*, *cdc6*, *cdc45*, *top2a*, *ttk*, *ccnb1* and *kif23* (Figure 6C). In this analysis, degree indicates network connectivity rather than regulatory importance.

Most candidates showed higher relative expression in BAE20 than in other groups, with lower or less consistent expression in BAE200 (Figure 6D). The resulting network prioritized candidates for future time-resolved studies of cell proliferation and developmental progression.

## 4. Discussion

Our results showed that TBBPA-BAE elicited selective, concentration-specific and stage-dependent responses rather than a uniform developmental syndrome. Using the zebrafish embryo-larval model, we found that different toxicity endpoints became responsive at different times, pointing to distinct windows of susceptibility during larval development. These non-monotonic patterns are especially informative, as the highest nominal concentration did not consistently produce the strongest responses. A simple ranking of concentrations therefore cannot adequately capture the divergent response patterns observed across endpoints. Together, the functional, morphological and molecular data indicate that the predominant biological responses to TBBPA-BAE exposure vary across early developmental stages.

Several early observations are most readily understood as shifts in developmental timing or function rather than signs of broad developmental failure. The hatching delay observed at 54 hpf had largely resolved by 72–96 hpf, favoring a transient change in hatching timing. Heart rate increased only in BAE0.2, indicating a concentration-specific cardiac response. Locomotor effects were most evident in BAE20 and BAE200, indicating a concentration-specific reduction in basal swimming performance, consistent with selective changes in locomotor function. This pattern accords broadly with reports of neurodevelopmental or systemic effects of TBBPA and related derivatives [5,6,7,8,9]. The coexistence of functional and biochemical changes during early development prompted closer consideration of redox balance and craniofacial cartilage at 5 dpf.

At 5 dpf, biochemical disturbance occurred alongside selective changes in craniofacial cartilage. H_2_O_2_ increased across the tested concentrations, while CAT, T-SOD and radical-scavenging measures followed different profiles. Such divergence is consistent with disturbed redox homeostasis rather than a coordinated increase in antioxidant capacity. Together, these changes indicate disturbed redox balance. At the same developmental stage, cartilage-positive area and selected dimensions changed, while angular measures and overall craniofacial organization were largely preserved. The phenotype is therefore better described as a localized cartilage alteration than as an overt craniofacial malformation. The concurrent redox and cartilage responses show that biochemical and structural processes were both sensitive at this stage [24,25].

The skeletal phenotype observed at 14 dpf was equally heterogeneous. After normalization to craniofacial area, mineralization remained increased in BAE2 and BAE20. The increase in TRAP-positive area was accompanied by higher *acp5a* expression across low-to-intermediate concentrations, providing complementary evidence of an osteoclast-associated response. Other markers were less uniform, with *nfatc1* and *ocstamp* responding mainly in BAE20 while *fosab* and *ctsk* showed no consistent pattern. The mixed *tnfsf11*, *tnfrsf11a* and *tnfrsf11b* profiles, including the pronounced OPG-encoding response in BAE20, suggested changes in transcriptional regulation rather than uniform activation of the RANKL–RANK–OPG system. Increased TRAP staining could reflect changes in remodeling activity, differentiation state or the cellular composition of cranial tissues. Mineralization itself is likely to integrate remodeling, matrix deposition, osteoblast function, growth and developmental timing, which may account for its non-monotonic pattern [24,26,27].

The 5 dpf transcriptome provides an early molecular reference for interpreting the developmental response. Recent comparative studies of bisphenol alternatives have shown that structurally related substitutes can differ markedly in embryonic toxicity, stress-related responses, and endocrine activity [28,29,30]. Redox-response-related expression patterns were considered alongside the measured biochemical redox disturbance, while cartilage- and matrix-related genes were relevant to the structural phenotype assessed at the same stage [27,31]. Inflammation/AP-1 signatures identified additional early stress-related themes. The later skeletal findings then prompted a phenotype-guided examination of cartilage-, matrix- and osteoclast-related transcripts in the earlier dataset. Changes in these gene sets at 5 dpf identified molecular processes potentially relevant to the later skeletal responses and provided a basis for time-resolved investigation. The concentration pattern also cautions against a linear interpretation because BAE20 produced the strongest early transcriptomic response, whereas some later endpoints were most responsive in BAE2. By contrast, several responses weakened or reversed in BAE200. This distinction makes BAE20 valuable for molecular follow-up while reinforcing that no single concentration ranked highest across all endpoints.

BAE20 also yielded the clearest molecular perturbation, making it the most informative concentration for the exploratory WGCNA/PPI analysis. Recent network-based high-throughput transcriptomics in zebrafish embryos similarly illustrates how concentration-dependent molecular signals can be mapped to phenotypic outcomes while retaining uncertainty [32]. The candidate network emphasized DNA replication, cell cycle and chromosome organization, processes associated with cell proliferation and developmental progression. Highly connected genes may therefore mark a proliferation-related program that is particularly responsive at 5 dpf. Node connectivity was used to identify candidates rather than to assign regulatory control. The resulting candidate set provides a focused starting point for follow-up studies at BAE20 and remains separate from the skeletal interpretation.

The multi-endpoint design allowed the developmental response to be examined from early function and redox balance through later skeletal outcomes. Because different developmental stages were assessed in separate cohorts and some endpoints used different exposure formats, the cross-stage patterns are interpreted as complementary responses rather than as a direct temporal sequence. Continuous exposure across two developmental stages revealed shifts in the dominant response with stage and concentration. Contemporary syntheses of skeletal-cell metabolism and osteoclast transcriptional control also emphasize that mineralization and remodeling reflect coordinated, cell-specific processes rather than a single molecular readout [33,34]. WGCNA was used as an exploratory analysis to prioritize treatment-associated candidate genes, and its interpretation is limited by the relatively small RNA-seq sample size. The nominal concentration series enabled comparison across a broad range, including BAE0.2, which was selected to approximate the upper end of reported aquatic concentrations, and higher groups used for hazard characterization [3]. Although aqueous concentrations and internal burdens were not measured, the predefined nominal series allowed biological responses to be compared among treatment groups under the same experimental conditions.

Taken together, these findings provide a broader view of the developmental responses associated with TBBPA-BAE exposure across multiple biological levels and developmental stages. The combined phenotypic, biochemical, and molecular results highlight redox disturbance and skeletal alterations as responsive components of TBBPA-BAE developmental toxicity, without implying a direct causal relationship between them. Future studies incorporating measured exposure concentrations and more detailed temporal and cell-specific analyses will help clarify how these responses are related.

## 5. Conclusions

Continuous TBBPA-BAE exposure induced selective, non-monotonic and stage-dependent developmental responses in zebrafish. Redox homeostasis was disrupted during early development, accompanied by changes in craniofacial cartilage and concentration-specific transcriptomic responses. At 14 dpf, TBBPA-BAE altered craniofacial mineralization and osteoclast-associated responses, indicating that skeletal development and remodeling are sensitive to developmental exposure. The 5 dpf transcriptome identified redox- and cartilage-related expression changes and molecular processes potentially relevant to the later skeletal responses, while the DNA replication/cell-cycle network provided additional candidates for time-resolved investigation. Overall, these findings identify early redox disturbance and stage-dependent skeletal alterations as two prominent components of the developmental response to TBBPA-BAE.

## Figures and Tables

**Figure 1:**
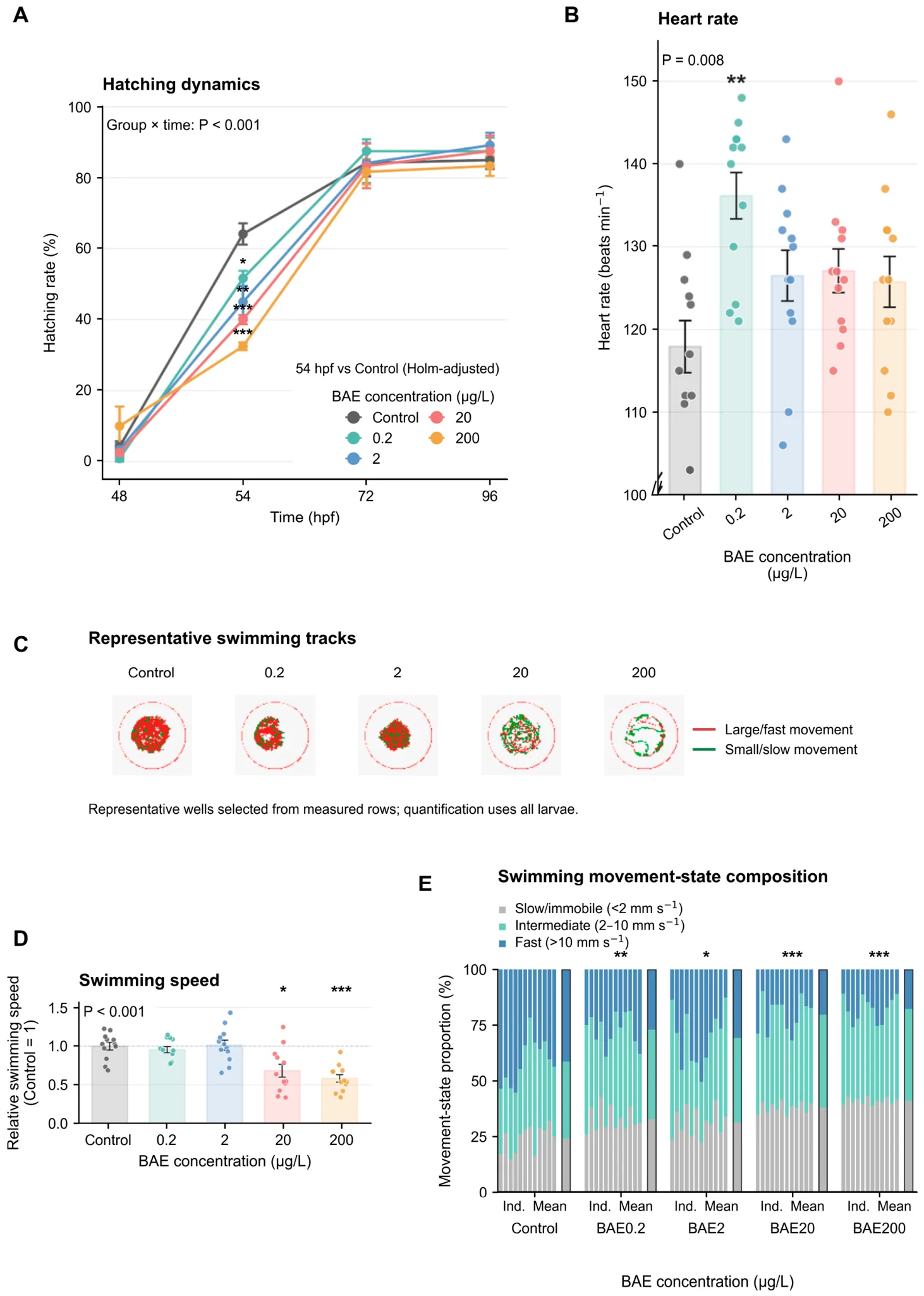
Effects of TBBPA-BAE exposure on developmental, cardiac and locomotor endpoints in zebrafish larvae. (**A**) Hatching dynamics at 48, 54, 72 and 96 hpf. Hatching was analyzed longitudinally using a binomial generalized estimating equation model accounting for repeated observations of the same embryos. The *p* value indicates the overall treatment × time interaction; asterisks indicate Holm-adjusted treatment-versus-control comparisons. (**B**) Heart rate at 96 hpf. The *p* value indicates the overall group effect; asterisks indicate Holm-adjusted comparisons with the control. (**C**) Representative swimming trajectories recorded during the 10 min basal locomotor assay. Red trajectories indicate large/fast movements, whereas green trajectories indicate small/slow movements. Representative wells were selected for visualization, while all measured larvae were included in the quantitative analyses. (**D**) Mean swimming speed during the 10 min recording period, expressed relative to the control mean. (**E**) Proportions of recording time classified as slow/immobile (<2 mm s^−1^), intermediate (2–10 mm s^−1^) or fast (>10 mm s^−1^) movement. The three movement states were analyzed jointly as compositional data. Asterisks indicate Holm-adjusted joint comparisons of the complete movement-state composition with the control. For panels (**A**–**E**), *n* = 12 individuals per treatment from three independent experimental runs (4 individuals/run). Each individual originated from a separate exposure well. Bars are presented as mean ± s.e.m. In panels (**B**,**D**), each point represents one independent exposure well containing one larva. In panel (**E**), thin stacked bars show the movement-state composition of individual larvae, whereas the wider bar labeled “Mean” shows the group mean composition. The *p* values shown in panels (**B**,**D**) indicate overall group effects. Asterisks indicate significant differences from the control after Holm adjustment: * *p* < 0.05, ** *p* < 0.01 and *** *p* < 0.001.

**Figure 2:**
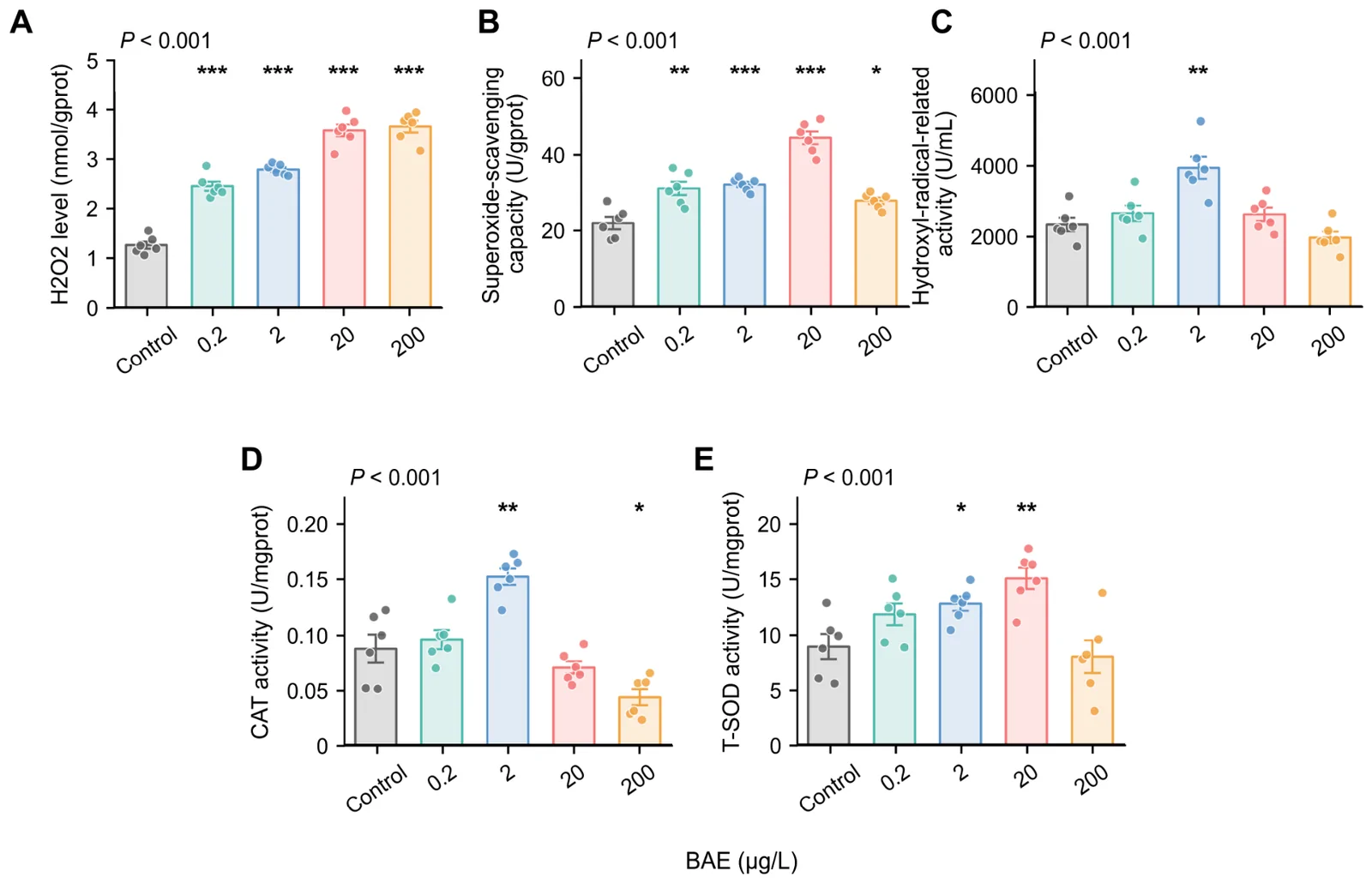
Redox-related endpoints in zebrafish larvae at 5 dpf after continuous exposure to 0.2, 2, 20 or 200 µg/L TBBPA-BAE. (**A**) H_2_O_2_ level, (**B**) superoxide-scavenging capacity, (**C**) hydroxyl-radical-related activity, (**D**) catalase (CAT) activity and (**E**) total superoxide dismutase (T-SOD) activity. For all parameters, *n* = 6 well-derived biological replicates per treatment from three independent experimental runs (2 wells/run). Each replicate consisted of 20 larvae pooled from one exposure well. Bars represent mean ± s.e.m., and each point represents one independent well-derived pooled sample. Upper-left *p* values denote overall group effects, and asterisks denote Dunnett-adjusted comparisons with the control (* *p* < 0.05, ** *p* < 0.01, *** *p* < 0.001).

**Figure 3:**
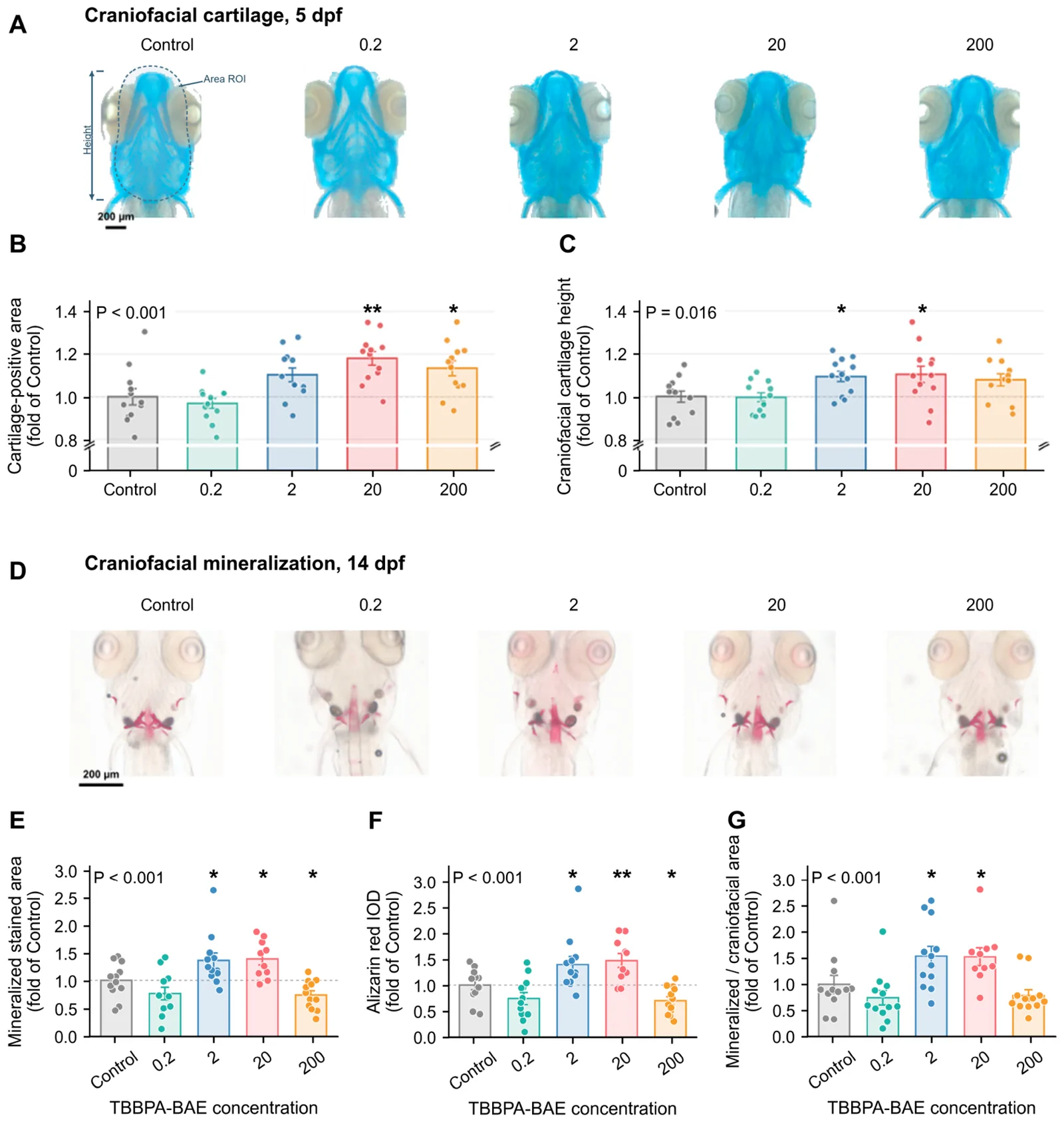
Effects of TBBPA-BAE exposure on craniofacial cartilage development and bone mineralization in zebrafish. (**A**) Representative ventral-view images of Alcian blue-stained craniofacial cartilage at 5 dpf. The dashed outline in the control image indicates the region of interest used to quantify cartilage-positive area, and the vertical line indicates craniofacial cartilage height. Scale bar, 200 μm. (**B**) Control-normalized cartilage-positive area. (**C**) Control-normalized craniofacial cartilage height. (**D**) Representative ventral-view images of Alizarin red-stained craniofacial mineralization at 14 dpf. Scale bar, 200 μm. (**E**) Control-normalized mineralized area. (**F**) Control-normalized integrated optical density (IOD) of Alizarin red staining. (**G**) Mineralized area normalized to the corresponding craniofacial area of each larva and expressed relative to the control-group mean. For panels (**B**,**C**,**E**–**G**), *n* = 12 larvae per treatment from three independent experimental runs (4 larvae/run), with each larva originating from a separate exposure well containing one larva. Bars represent mean ± s.e.m., and each point represents one larva from an independent exposure well. The *p* values shown in the panels indicate overall differences among groups. Asterisks indicate significant differences from the control after Holm adjustment: * *p* < 0.05, ** *p* < 0.01.

**Figure 4:**
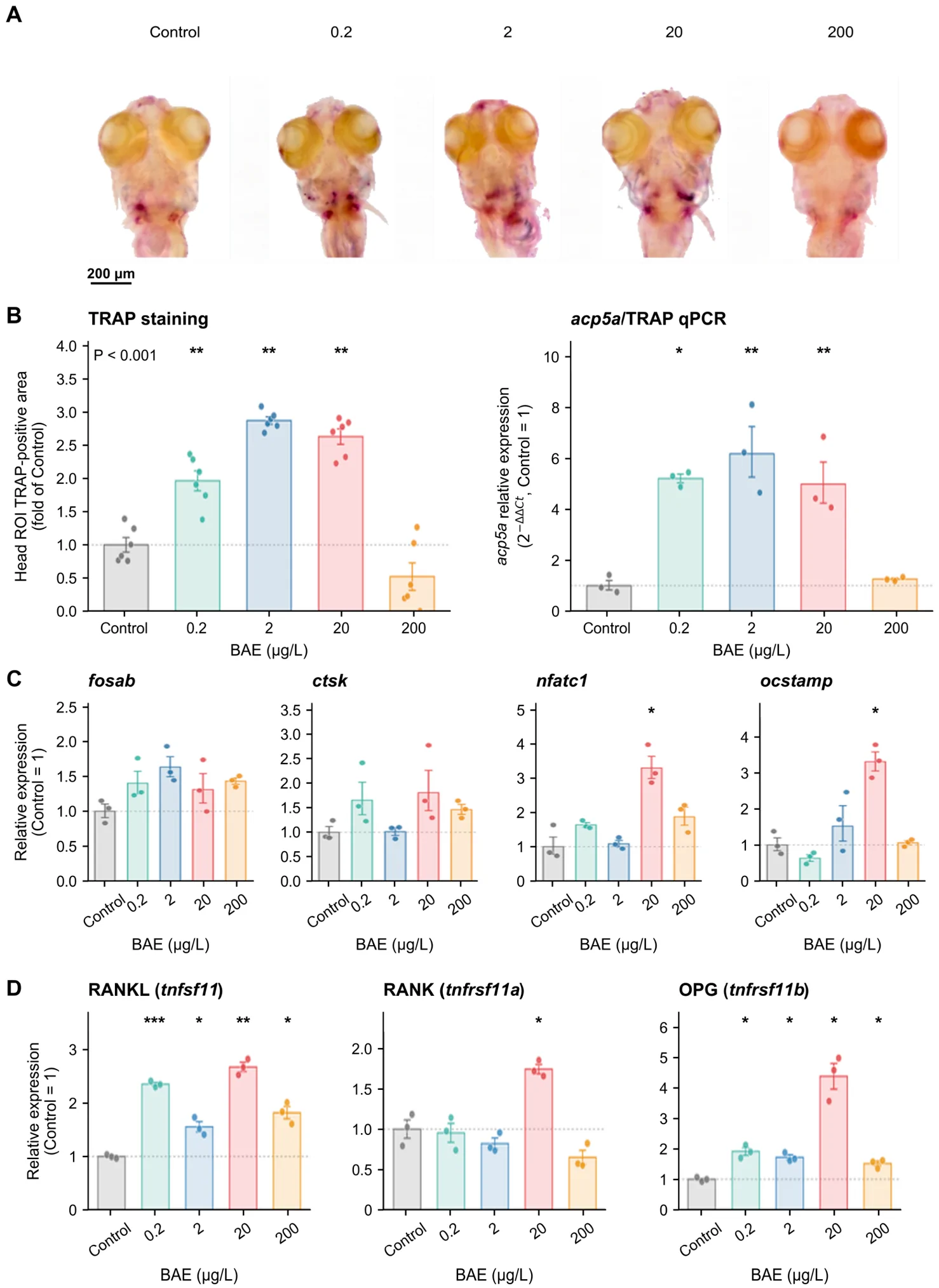
Osteoclast-associated staining and targeted transcript expression at 14 dpf after continuous exposure to 0.2, 2, 20 or 200 µg/L TBBPA-BAE. (**A**) Representative cranial TRAP staining. (**B**) Cranial TRAP-positive area and *acp5a* expression. (**C**) Expression of *fosab, ctsk, nfatc1* and *ocstamp*. (**D**) Expression of *tnfsf11*/*RANKL*, *tnfrsf11a*/*RANK* and *tnfrsf11b*/*OPG*. For TRAP-positive area, *n* = 6 larvae per treatment from three independent experimental runs (2 larvae/run). For RT-qPCR, *n* = 3 pooled biological replicates per treatment, with one pooled replicate generated from each independent experimental run. Each RT-qPCR biological replicate contained 20 pooled larvae. Bars represent mean ± s.e.m. For TRAP-positive area, each point represents one independent exposure well containing one larva; for RT-qPCR, each point represents one pooled biological replicate. The upper-left *p* value in the TRAP-positive-area panel denotes the permutation-based omnibus test. RT-qPCR comparisons with the control were conducted on ΔCt values using two-sided Welch’s *t*-tests with Holm correction across the four comparisons within each gene. Asterisks denote adjusted comparisons with the control (* *p* < 0.05, ** *p* < 0.01, *** *p* < 0.001).

**Figure 5:**
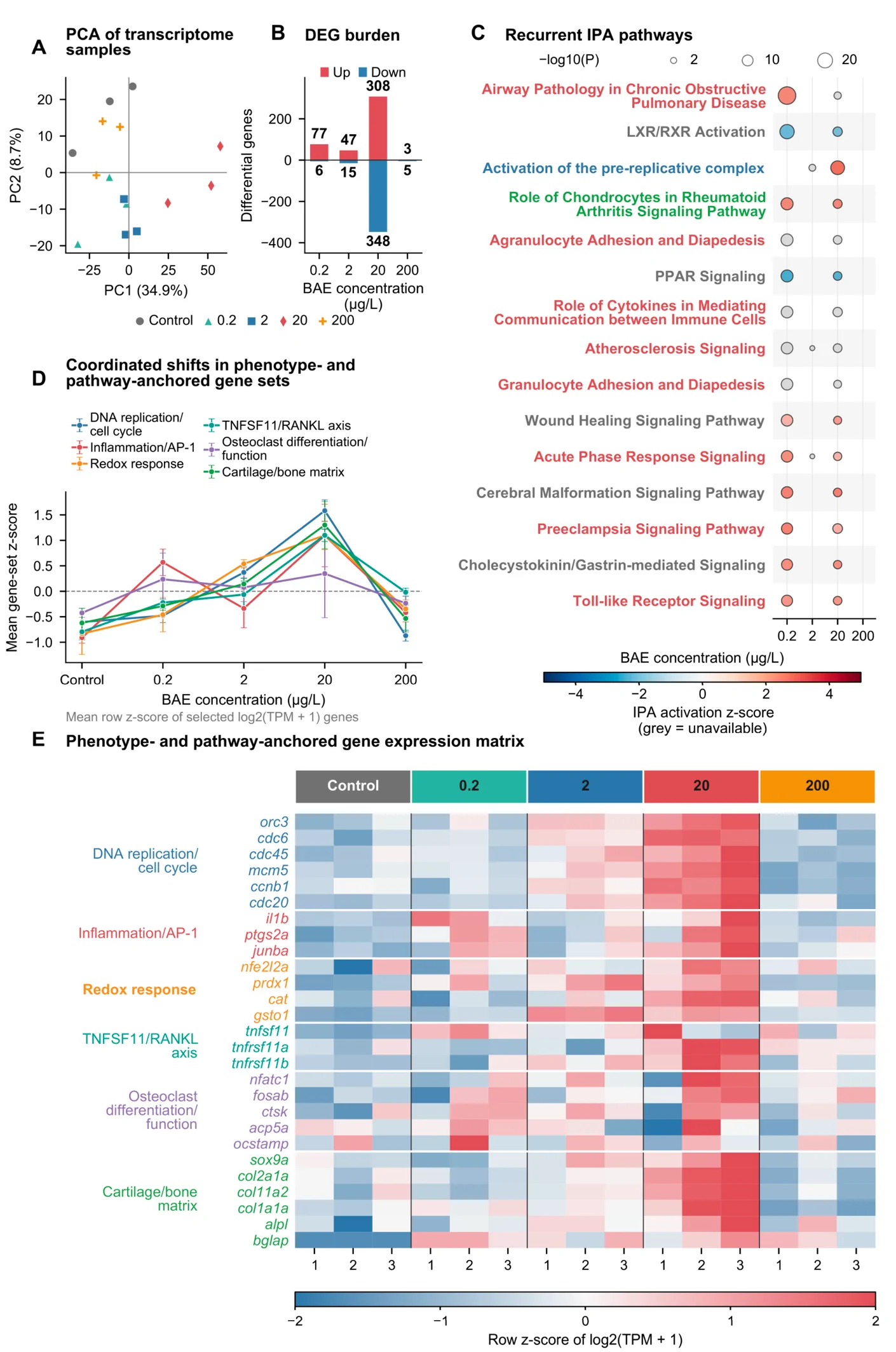
Transcriptomic responses at 5 dpf after continuous TBBPA-BAE exposure. (**A**) principal component analysis of the RSEM-derived gene-level TPM matrix, (**B**) formal differentially expressed gene (DEG) counts per BAE group relative to the control, defined using a Benjamini–Hochberg-adjusted *p* value < 0.05 and an absolute fold change of at least 1.5 (|log_2_FC| ≥ 0.585), (**C**) the 15 highest-ranked recurrent exploratory IPA pathways (present in at least two exposure groups), ranked by the minimum within-export Benjamini–Hochberg FDR across concentrations and then by the maximum −log_10_(P), with bubble size denoting −log_10_(P), color denoting the IPA activation z-score and gray denoting unavailable z-scores, (**D**) group-level mean ± s.e.m. row z-scores for selected phenotype- and pathway-anchored gene sets and (**E**) sample-level row z-scores of log_2_(TPM + 1) for selected genes; RNA-seq included three pooled biological replicates per treatment, with one replicate generated from each of three independent experimental runs and 100 larvae pooled per replicate. Disease-labeled pathways are interpreted as molecular signatures rather than diagnoses, and targeted gene-set comparisons are descriptive and hypothesis-generating. Row z-scores represent within-gene standardized expression patterns and are not control-normalized values.

**Figure 6:**
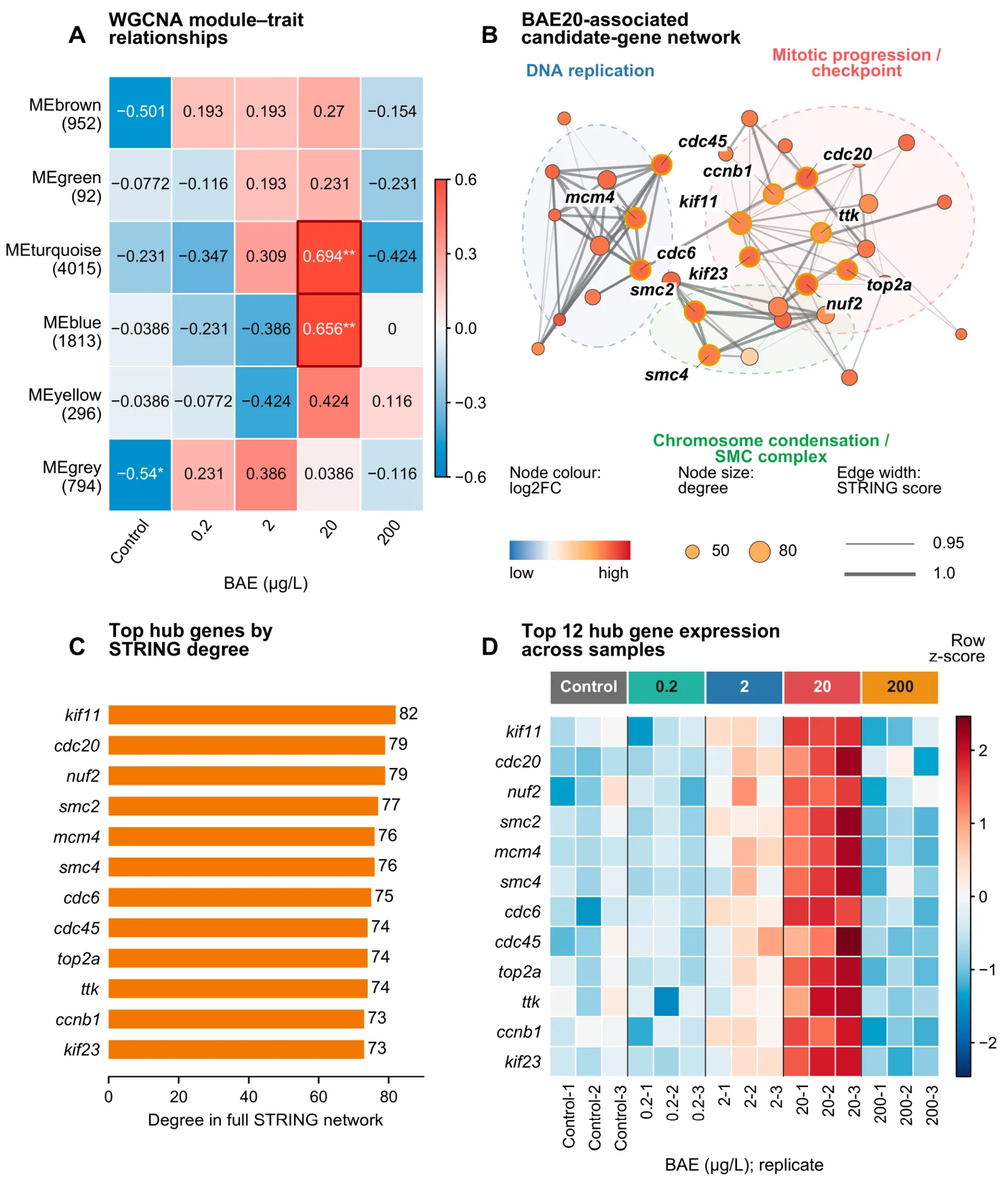
Exploratory BAE20-associated WGCNA/PPI candidate network. (**A**) WGCNA module–trait relationships from all 15 RNA-seq samples based on Spearman correlations between module eigengenes and each treatment group; *p* values are nominal, (**B**) the STRING protein–protein interaction network obtained by intersecting positively associated modules with BAE20 differentially expressed genes, where node color denotes log_2_FC, node size denotes STRING degree and edge width denotes the STRING combined score, (**C**) degree-ranked candidate genes in the retained network and (**D**) sample-level row z-scores of log_2_(TPM + 1) for the top 12 candidates. Row z-scores represent within-gene standardized expression patterns derived from log_2_(TPM + 1) values and are not normalized to the control group. In panel (**A**), asterisks denote nominal module–trait correlation significance (* *p* < 0.05, ** *p* < 0.01).

## Data Availability

The raw RNA-sequencing data generated in this study have been deposited in the NCBI Sequence Read Archive (SRA) under BioProject accession PRJNA1512095 (SRA runs: SRR40145874–SRR40145888) and have been released for public access. Additional data supporting the findings of this study are provided in the article and its Supplementary Materials.

## Supplementary Materials

The following supporting information can be downloaded at: https://www.mdpi.com/article/10.3390/antiox15101242/s1, Table S1: Water-quality parameters in the supplementary verification experiment; Table S2: Endpoint-specific exposure and replication design; Table S3: Details of the redox-related biochemical assays; Table S4: RT-qPCR primer sequences; Table S5: RT-qPCR quality control and plate allocation; Table S6: RNA-seq reproducibility and sample QC; Table S7: IPA input, identifier mapping and recurrence audit; Table S8: Representative gene-set membership and classification rationale; Table S9: Leave-one-sample-out sensitivity analysis of WGCNA module–treatment trait relationships; Table S10: Hypothesis families and interpretation; Table S11: RANKL/OPG mRNA ratios; Figure S1: Additional craniofacial morphometric results and representative Alcian blue measurement landmarks; Figure S2: RT-qPCR primer standard curves and amplification efficiencies; Figure S3: Representative RT-qPCR melting-peak profiles; Figure S4: Selected KEGG GSEA pathways across TBBPA-BAE concentrations; Figure S5: Craniofacial area and its relationship with mineralization at 14 dpf; Supplementary Dataset S1: Differential-expression, GSEA, IPA, WGCNA and STRING supporting data.
