# Supplementary material for: Developmental Toxicity of Tetrabromobisphenol A Bis(allyl ether) in Zebrafish: Redox Disturbance and Osteoclast-Associated Skeletal Responses
Source: Antioxidants (Basel). 2026 Sep 26;15(10):1242. doi: 10.3390/antiox15101242 (PMC13700801; doi:10.3390/antiox15101242)
Supplement: Supplementary file 1 [file antioxidants-15-01242-s001.zip › Supplementary_Information.pdf]

Supplementary Information

Developmental Toxicity of Tetrabromobisphenol A Bis(allyl ether) in Zebrafish: Redox Disturbance and Osteoclast-Associated Skeletal Responses

Lele Fu [1], Hai Xu [2], Qing Wei [1], Yungu Zheng [1], Ling Cai [3,*] and Ming Yang [1,*]

[1] School of Environmental and Chemical Engineering, Shanghai University, Shanghai 200444, China

[2] School of Environment and Safety Engineering, Jiangsu University, Zhenjiang 212013, China

[3] Key Laboratory of Marine Ecological Conservation and Restoration, Third Institute of Oceanography, Ministry of Natural Resources, Xiamen 361005, China

* Correspondence: cailing@tio.org.cn (L.C.); mingyang@shu.edu.cn (M.Y.)

Contents

Supplementary Table S1. Water-quality parameters in the supplementary verification experiment

Supplementary Table S2. Endpoint-specific exposure and replication design

Supplementary Table S3. Details of the redox-related biochemical assays

Supplementary Table S4. RT–qPCR primer sequences

Supplementary Table S5. RT-qPCR quality control and plate allocation

Supplementary Table S6. RNA-seq reproducibility and sample QC

Supplementary Table S7. IPA input, identifier mapping and recurrence audit

Supplementary Table S8. Representative gene-set membership and classification rationale

Supplementary Table S9. Leave-one-sample-out sensitivity analysis of WGCNA module – treatment trait relationships

Supplementary Table S10. Hypothesis families and interpretation

Supplementary Table S11. RANKL/OPG mRNA ratios

Supplementary Figure S1. Additional craniofacial morphometric results and representative Alcian blue measurement landmarks

Supplementary Figure S2. RT-qPCR primer standard curves and amplification efficiencies

Supplementary Figure S3. Representative RT-qPCR melting-peak profiles

Supplementary Figure S4. Selected KEGG GSEA pathways across TBBPA-BAE concentrations

Supplementary Figure S5. Craniofacial area and its relationship with mineralization at 14 dpf

Supplementary Dataset S1. Differential-expression, GSEA, IPA, WGCNA and STRING supporting data

Supplementary Table S1 | Water-quality parameters in the supplementary verification experiment

Group	Temperature (°C)	pH	Dissolved oxygen (mg/L)	Ammonia nitrogen (mg/L)
Control	28	7.38	8.17	0.3141
BAE0.2	28	7.41	8.26	0.3607
BAE2	28	7.46	8.15	0.3374
BAE20	28	7.54	8.23	0.3316
BAE200	28	7.53	8.11	0.3374

Note. Water-quality measurements were obtained in a supplementary verification experiment conducted under the same exposure conditions as the original study.

Supplementary Table S2 | Endpoint-specific exposure and replication design

Well counts refer to the stated design; pooling does not increase the number of independent assayed samples.

Endpoint	Stage	Independent runs	Spawning events	Exposure format	Plate structure per treatment per run	Stocking density	Treatment preparation	Biological unit	Final sample size
Hatching	48/54/72/96 hpf	3	3	Individual exposure	1 twelve-well plate/treatment/run	1 embryo/well; 5 mL/well	Freshly and independently prepared at each 24-h renewal	Individual embryo in an independent exposure well	*n* = 12 embryos (4 embryos/run × 3 runs)
Heart rate	96 hpf	3	3	Individual exposure	1 twelve-well plate/treatment/run	1 larva/well; 5 mL/well	Same	Individual larva in an independent exposure well	*n* = 12 larvae (4 larvae/run × 3 runs)
Locomotor behavior	120 hpf	3	3	Individual exposure	1 twelve-well plate/treatment/run	1 larva/well; 5 mL/well	Same	Individual larva in an independent exposure well	*n* = 12 larvae (4 larvae/run × 3 runs)
Cartilage	5 dpf	3	3	Individual exposure	1 twelve-well plate/treatment/run	1 larva/well; 5 mL/well	Same	Individual larva in an independent exposure well	*n* = 12 larvae (4 larvae/run × 3 runs)
Mineralization	14 dpf	3	3	Individual exposure	1 twelve-well plate/treatment/run	1 larva/well; 5 mL/well	Same	Individual larva in an independent exposure well	*n* = 12 larvae (4 larvae/run × 3 runs)
TRAP staining	14 dpf	3	3	Individual exposure	1 twelve-well plate/treatment/run	1 larva/well; 5 mL/well	Same	Individual larva in an independent exposure well	*n* = 6 larvae (2 larvae/run × 3 runs)
Redox assays	5 dpf	3	3	Group exposure	1 six-well plate/treatment/run	20 larvae/well; 10 mL/well	Same	One well-derived pooled sample containing 20 larvae	*n* = 6 pooled biological replicates (2 wells/run × 3 runs)
RT-qPCR	14 dpf	3	3	Group exposure	1 six-well plate/treatment/run	20 larvae/well; 10 mL/well	Same	One pooled biological sample/run	*n* = 3 pooled biological replicates (1 pool/run × 3 runs)
RNA-seq	5 dpf	3	3	Group exposure	2 six-well plates/treatment/run (10 wells sampled)	20 larvae/well; 10 mL/well	Same	One pooled biological sample/run	*n* = 3 pooled biological replicates (1 pool/run × 3 runs)

Note: Different endpoints were conducted using separate experimental cohorts and were not necessarily derived from the same spawning events. Each endpoint was performed in three independent experimental runs, with each run initiated from an endpoint-specific spawning event. Within each run, all treatment groups were derived from the same spawning batch and processed in parallel. For individual-level endpoints with *n* = 12, four larvae were analyzed per treatment in each run; for TRAP staining, two larvae were analyzed per treatment in each run. For redox assays, two independent exposure wells per treatment were sampled in each run, with 20 larvae pooled from each well to form one biological.

replicate. For RT-qPCR and RNA-seq, one pooled biological replicate per treatment was generated in each run. Exposure solutions for each treatment were freshly and independently prepared at every 24-h renewal.

Supplementary Table S3 | Details of the redox-related biochemical assays

Note: All kits were obtained from Nanjing Jiancheng Bioengineering Institute (Nanjing, China). For each treatment, six well-derived biological replicates were analyzed across three independent experimental runs (2 wells/run × 3 runs). Each biological replicate consisted of 20 larvae pooled from one exposure well. Assays were performed according to the

Parameter	Assay kit / catalog no.	Assay principle	Detection wavelength	Sample type	Reporting
H_2O_2	Hydrogen Peroxide Assay Kit, A064-1-1	H_2O_2 reacts with molybdate to form a colored complex, and the amount of H_2O_2 is determined colorimetrically	405 nm	Homogenate from 20 larvae pooled from one exposure well	H_2O_2 level
Superoxide-scavenging capacity	Inhibition and Production of Superoxide Anion Assay Kit, A052-1-1	Superoxide is generated using a xanthine/xanthine-oxidase reaction system; inhibition of the color reaction reflects superoxide-scavenging capacity	550 nm	Same	Superoxide-scavenging capacity
Hydroxyl-radical-related activity	Hydroxyl Radical Assay Kit, A018-1-1	Hydroxyl radicals are generated by the Fenton reaction and quantified using a colorimetric Griess-based reaction	550 nm	Same	Hydroxyl-radical-related activity
Catalase (CAT)	Catalase Assay Kit, visible-light/ammonium molybdate method, A007-1-1	CAT decomposes H_2O_2; the remaining H_2O_2 reacts with ammonium molybdate to form a yellow complex	405 nm	Same	CAT activity
Total superoxide dismutase (T-SOD)	T-SOD Assay Kit, hydroxylamine method, A001-1	Superoxide generated by the xanthine-oxidase system reacts with hydroxylamine; inhibition of the color-forming reaction is used to calculate T-SOD activity	550 nm	Same	T-SOD activity

manufacturer’s instructions

Supplementary Table S4 | RT–qPCR primers

Gene	Target/name	Gene accession no. (Ensembl gene ID)	Forward primer (5′–3′)	Reverse primer (5′–3′)	Length, F/R (nt)	Role in this study	Efficiency (%)	R^2
actb	β-actin	**ENSDARG00000037746**	CGAGCGTGGCTACAGCTTCA	GACCGTCAGGCAGCTCATAG	20/20	Reference gene; internal control	94.7	0.9955
acp5a	TRAP	**ENSDARG00000019763**	CCATGTAGGAAACGTCAAAGC	GAATGCGGAAGTTCATCTCAT	21/21	TRAP-associated activity	98.1	0.9978
fosab	AP-1/c-Fos-related	**ENSDARG00000031683**	GGAGCAAAGACCTCCAACAAT	CTTGTTTCGTTCACGACGTA	21/20	Osteoclast/AP-1 response	99.5	0.9998
ctsk	Cathepsin K	**ENSDARG00000040251**	ACCCAAACTGCAACAAGG	TAGCCCTTCTTTCCCCAC	18/18	Resorptive function	98.2	0.9997
nfatc1	NFATc1	**ENSDARG00000036168**	CCGAGAGCAACATGAGAGC	AGCTCGATGTCTGAGTTACGC	19/21	Osteoclast differentiation	101.4	0.9998
ocstamp	OC-STAMP	**ENSDARG00000022139**	TCAGGTGGTCCTTGGATTTC	AATGGGTACTTTTGTTCCAACCT	20/23	Fusion/multinucleation	101.6	0.9999
tnfsf11	RANKL	**ENSDARG00000068141**	TAGTGTGGCGATTCTGTTGC	ATTGGAAGGTGAGCTGATGG	20/20	RANKL–RANK–OPG axis	93.8	0.9979
tnfrsf11a	RANK	**ENSDARG00000087804**	AAGTGGACAGATTGTAAAGCTAT	GCCACCTGATGAGGTTTCAGCAC	23/23	RANKL–RANK–OPG axis	100.8	0.9998
tnfrsf11b	OPG	**ENSDARG00000098377**	GGCGTCTGAAGAAACCTCTG	GCAGGATTGGGATGCAGTAT	20/20	RANKL–RANK–OPG axis	100.2	0.9999

Note. Primer sequences are reported 5′ to 3′. Gene accession/identifier is reported as the Ensembl gene ID for *Danio rerio*. actb was used as the reference gene. Gene symbols follow zebrafish nomenclature. Primer standard curves and amplification efficiencies are shown in Supplementary Figure S2.

Supplementary Table S5 | RT-qPCR quality control and plate allocation

Parameter	Information
Biological replication	Three independent biological replicates per treatment
Technical replication	Three technical replicates per biological sample and gene
Melting-curve analysis	A single dominant melting peak was observed for each primer pair
No-template control	Included
Exclusion criterion	A technical replicate was considered discordant when its Cq differed by >0.5 cycles from the other two concordant wells
Exclusions	None
Plate arrangement	All treatment groups and biological replicates for each gene were analyzed on the same plate
Primer efficiency	93.8 – 101.6%; R^2 = 0.9955 – 0.9999; evaluated using five-fold serial dilutions of cDNA
Reference-gene assessment (actb)	actb Cq values were assessed using three biological replicates per group, with each biological replicate represented by the mean of three technical replicates. Group values (mean ± SD) were 21.38 ± 0.42 (Control), 21.35 ± 0.12 (BAE0.2), 21.64 ± 0.36 (BAE2), 21.48 ± 0.17 (BAE20), and 21.43 ± 0.67 (BAE200). The maximum difference among group means was 0.29 cycles, and no significant overall difference was detected among groups (Welch's ANOVA, P = 0.777).
No-RT control	Not included in the original experiment

Supplementary Table S6 | RNA-seq reproducibility and sample QC

A. Experimental design, sample preparation and bioinformatics

Section	Parameter	Information
**Experimental design**	Samples	15 libraries; 5 groups × 3 biological replicates
	Biological material	Whole zebrafish larvae at 5 dpf
	Biological replicate	100 larvae pooled from 10 group-exposure wells
**RNA preparation**	RNA extraction	TRIzol followed by RNeasy Mini Kit purification with on-column DNase treatment
	RNA quality assessment	NanoDrop 2000, agarose-gel electrophoresis and Agilent 5300
	RNA input requirement	1 μg total RNA; ≥30 ng/μL; RQN >6.5; OD260/280 = 1.8–2.2
**Library preparation**	mRNA enrichment	Poly(A) mRNA enriched using oligo(dT) magnetic beads
	Fragmentation	Approximately 300 bp
	Library workflow	cDNA synthesis, end repair, A-tailing, adapter ligation and PCR enrichment
	Commercial library kit	Not specified in archived sequencing-provider report
	Strandedness	Not specified in archived sequencing-provider report
**Sequencing**	Platform	Illumina NovaSeq; exact model not specified
	Read layout	Paired-end, 2 × 151 bp
	Total clean data	90.06 Gb
**Bioinformatics**	Read quality control	SeqPrep and fastp v0.23.4
	Filtering thresholds	Not specified in archived sequencing-provider report
	Reference species	*Danio rerio*
	Genome build / annotation	Exact genome build and annotation release not specified in archived report
	Alignment	HISAT2 v2.2.1
	Quantification	RSEM; gene-level read counts and TPM
	Differential expression	DESeq2 v1.42.0
	Formal DEG criterion	BH-adjusted P < 0.05 and|FC|≥ 1.5 (|$\log_2$FC|≥ 0.585)
	Gene pre-filtering	No additional criterion documented in archived provider report
	Detected genes	27,228 expressed genes
**Batch / archive**	Technical batch information	Separate library/lane batch identifiers not provided in archived report
	Public archive	BioProject PRJNA1512095; SRR40145874–SRR40145888

RNA-seq experimental details and sample quality metrics

B. Individual-library sequencing and mapping quality

Sample	Group	Raw reads	Clean reads	Clean data (Gb)	Q30 (%)	GC (%)	Total mapping (%)	Unique mapping (%)
SC_1	Control	38,973,396	38,688,272	5.812	96.72	48.23	92.54	87.76
SC_2	Control	39,632,816	39,349,112	5.911	96.71	48.61	93.13	88.22
SC_3	Control	38,334,866	37,997,876	5.710	96.61	47.99	92.25	87.29
BAE02_1	BAE0.2	36,586,938	36,300,528	5.461	96.77	48.41	93.06	88.14
BAE02_2	BAE0.2	40,464,024	40,163,692	6.042	96.78	47.99	92.59	87.87
BAE02_3	BAE0.2	42,098,798	41,789,646	6.285	96.79	48.24	92.79	87.92
BAE2_1	BAE2	39,676,140	39,433,788	5.932	96.79	48.20	92.72	87.93
BAE2_2	BAE2	47,312,084	47,002,214	7.070	96.79	49.09	93.43	88.54
BAE2_3	BAE2	41,034,830	40,749,778	6.127	96.76	48.76	93.13	88.26
BAE20_1	BAE20	38,763,518	38,502,392	5.789	96.72	48.60	92.92	88.24
BAE20_2	BAE20	43,453,538	43,157,596	6.492	96.88	48.03	92.51	87.69
BAE20_3	BAE20	39,503,042	39,239,086	5.900	96.76	48.39	92.63	87.99
BAE200_1	BAE200	40,780,304	40,455,716	6.084	96.89	48.25	92.86	87.97
BAE200_2	BAE200	37,522,304	37,228,420	5.597	96.68	47.90	92.41	87.65
BAE200_3	BAE200	39,284,376	38,936,964	5.847	96.66	48.03	92.48	87.77

Abbreviations: dpf, days post-fertilization; TPM, transcripts per million; DEG, differentially expressed gene; BH, Benjamini–Hochberg; FC, fold change; RQN, RNA quality number.

Note. Fifteen RNA-seq libraries were analyzed, with three biological replicates per treatment. Sequencing was performed using paired-end 151-bp reads on an Illumina NovaSeq platform. Items reported as “not specified” were unavailable in the archived sequencing-provider report and were therefore not inferred from generic workflows.

Supplementary Table S7 | IPA input, identifier mapping and recurrence audit

Contrast	Formal DEGs	Exploratory IPA input	Successfully mapped genes	ENSDARG identifiers	Mapping success (%)
BAE0.2	83	378	276	102	73.0
BAE2	62	351	245	106	69.8
BAE20	656	1182	971	211	82.1
BAE200	8	200	140	60	70.0

Note. Formal DEGs were defined using a Benjamini–Hochberg-adjusted $P < 0.05$ and an absolute fold change ≥1.5. Genes used for exploratory IPA were selected using nominal DESeq2 $P < 0.05$ and the same fold-change threshold. Zebrafish genes were converted to human orthologs where possible before IPA analysis. Mapping success was calculated as the number of successfully mapped genes divided by the exploratory IPA input for each exposure group.

C.

Supplementary Table S8 | Representative gene sets

Representative gene set	Gene	Ensembl gene ID	Anchor	Classification rationale
DNA replication/cell cycle	*orc3*	ENSDARG00000008805	Pathway-anchored	Origin-recognition-complex subunit representing replication-origin licensing.
DNA replication/cell cycle	*cdc6*	ENSDARG00000009942	Pathway-anchored	Replication-licensing factor required for pre-replication-complex assembly.
DNA replication/cell cycle	*cdc45*	ENSDARG00000043720	Pathway-anchored	DNA-replication-initiation factor that activates the replicative helicase complex.
DNA replication/cell cycle	*mcm5*	ENSDARG00000019507	Pathway-anchored	MCM helicase component included as a marker of DNA-replication machinery.
DNA replication/cell cycle	*ccnb1*	ENSDARG00000051923	Pathway-anchored	Cyclin B1 representing G2/M cell-cycle progression.
DNA replication/cell cycle	*cdc20*	ENSDARG00000100741	Pathway-anchored	Mitotic-transition regulator and APC/C co-activator.
Inflammation/AP-1	*il1b*	ENSDARG00000098700	Pathway/early-stress-anchored	Pro-inflammatory cytokine linked to recurrent cytokine and inflammatory signatures.
Inflammation/AP-1	*ptgs2a*	ENSDARG00000004539	Pathway/early-stress-anchored	Cyclooxygenase-related inflammatory-response gene involved in prostaglandin synthesis.
Inflammation/AP-1	*junba*	ENSDARG00000074378	Pathway/early-stress-anchored	AP-1-family transcription-factor component representing stress/inflammatory transcription.
Redox response	*nfe2l2a*	ENSDARG00000042824	Phenotype-anchored	Nrf2-family redox-response regulator anchored to the measured redox endpoints.
Redox response	prdx1	ENSDARG00000058734	Phenotype-anchored	Peroxiredoxin representing peroxide detoxification and redox homeostasis.
Redox response	cat	ENSDARG00000104702	Phenotype-anchored	Catalase linking transcript abundance to the measured catalase/redox phenotype.
Redox response	*gsto1*	ENSDARG00000022183	Phenotype-anchored	Glutathione-transferase-family gene representing glutathione-dependent detoxification.
TNFSF11/RANKL axis	*tnfsf11*	ENSDARG00000068141	Phenotype-anchored	Encodes RANKL, the ligand component of the measured RANKL–RANK–OPG axis.
TNFSF11/RANKL axis	*tnfrsf11a*	ENSDARG00000087804	Phenotype-anchored	Encodes RANK, the cognate receptor in the measured axis.
TNFSF11/RANKL axis	*tnfrsf11b*	ENSDARG00000098377	Phenotype-anchored	Encodes OPG, the decoy receptor used in the mRNA-based RANKL/OPG index.
Osteoclast differentiation/function	*nfatc1*	ENSDARG00000036168	Phenotype-anchored	Transcriptional regulator widely used as an osteoclast-differentiation marker.
Osteoclast differentiation/function	*fosab*	ENSDARG00000031683	Phenotype-anchored	c-Fos/AP-1-related factor included for osteoclast-associated differentiation.
Osteoclast differentiation/function	*ctsk*	ENSDARG00000040251	Phenotype-anchored	Cathepsin-K protease included as a resorptive-function marker.
Osteoclast differentiation/function	*acp5a*	ENSDARG00000019763	Phenotype-anchored	TRAP-associated gene linked directly to the TRAP histochemical endpoint.

Representative gene set	Gene	Ensembl gene ID	Anchor	Classification rationale
Osteoclast differentiation/function	*ocstamp*	ENSDARG00000022139	Phenotype-anchored	Osteoclast-stimulatory transmembrane protein included as a cell-fusion marker.
Cartilage/bone matrix	*sox9a*	ENSDARG00000003293	Phenotype-anchored	Chondrogenic transcription factor linked to the cartilage phenotype domain.
Cartilage/bone matrix	*col2a1a*	ENSDARG00000069093	Phenotype-anchored	Type-II collagen representing cartilage extracellular matrix.
Cartilage/bone matrix	*col11a2*	ENSDARG00000012422	Phenotype-anchored	Type-XI collagen included as a cartilage-matrix structural component.
Cartilage/bone matrix	*col1a1a*	ENSDARG00000012405	Phenotype-anchored	Type-I collagen representing bone/extracellular-matrix deposition.
Cartilage/bone matrix	*alpl*	ENSDARG00000015546	Phenotype-anchored	Alkaline-phosphatase gene included as a mineralisation-associated marker.
Cartilage/bone matrix	*bglap*	ENSDARG00000058414	Phenotype-anchored	Osteocalcin gene included as a marker of mineralised bone matrix.

Note. The phenotype-guided gene sets were curated post hoc using predefined biological classification criteria and were analysed separately from the WGCNA modules. The DNA-replication/cell-cycle set was pathway anchored. Inclusion in these sets does not establish pathway activation, regulation or causality. Four sets were linked to measured or subsequently assessed phenotype domains, whereas the inflammation/AP-1 set represented an additional pathway-anchored early-stress category.

Supplementary Table S9. Leave-one-sample-out sensitivity analysis of WGCNA module – treatment trait relationships

Supplementary Table S9A. MEturquoise

Omitted sample	Treatment group	Control	BAE0.2	BAE2	BAE20	BAE200
SC_1	Control	−0.2532	−0.3671	0.2807	**0.7125**	−0.4102
SC_2	Control	0.0000	−0.4534	0.2807	**0.7125**	−0.5398
SC_3	Control	−0.3545	−0.3239	0.3239	**0.7125**	−0.4102
BAE02_1	BAE0.2	−0.2807	−0.2025	0.2807	**0.7125**	−0.5398
BAE02_2	BAE0.2	−0.2375	−0.4051	0.2807	**0.7125**	−0.4102
BAE02_3	BAE0.2	−0.2807	−0.3038	0.2807	**0.7125**	−0.4534
BAE2_1	BAE2	−0.2375	−0.3239	0.3038	**0.7125**	−0.4102
BAE2_2	BAE2	−0.1943	−0.3239	0.2532	**0.7125**	−0.4102
BAE2_3	BAE2	−0.1943	−0.3239	0.2532	**0.7125**	−0.4102
BAE20_1	BAE20	−0.1943	−0.3239	0.4102	**0.6076**	−0.4102
BAE20_2	BAE20	−0.1943	−0.3239	0.4102	**0.6076**	−0.4102
BAE20_3	BAE20	−0.1943	−0.3239	0.4102	**0.6076**	−0.4102
BAE200_1	BAE200	−0.2807	−0.4102	0.2807	**0.7125**	−0.3545
BAE200_2	BAE200	−0.2807	−0.3671	0.2807	**0.7125**	−0.4051
BAE200_3	BAE200	−0.2807	−0.4102	0.2807	**0.7125**	−0.3545

Supplementary Table S9B. MEblue

Omitted sample	Treatment group	Control	BAE0.2	BAE2	BAE20	BAE200
SC_1	Control	−0.1013	−0.2375	−0.3671	**0.6693**	0.0216
SC_2	Control	0.2532	−0.3239	−0.4966	**0.6693**	−0.0648
SC_3	Control	−0.2532	−0.1943	−0.3671	**0.7125**	0.0648
BAE02_1	BAE0.2	−0.0648	−0.0506	−0.4966	**0.6693**	−0.0648
BAE02_2	BAE0.2	−0.0216	−0.3545	−0.3671	**0.6693**	0.0216
BAE02_3	BAE0.2	−0.0648	−0.2025	−0.4102	**0.6693**	−0.0216
BAE2_1	BAE2	−0.0648	−0.2375	−0.4051	**0.6693**	−0.0216
BAE2_2	BAE2	−0.0648	−0.2807	−0.3038	**0.6693**	−0.0648
BAE2_3	BAE2	−0.0648	−0.2807	−0.3038	**0.6693**	−0.0648
BAE20_1	BAE20	−0.0216	−0.1943	−0.3671	**0.6076**	0.0648
BAE20_2	BAE20	0.0216	−0.1943	−0.3671	**0.5570**	0.0648
BAE20_3	BAE20	0.0216	−0.1943	−0.3671	**0.5570**	0.0648
BAE200_1	BAE200	−0.0648	−0.2807	−0.4102	**0.6693**	0.1013
BAE200_2	BAE200	−0.0216	−0.1943	−0.3671	**0.6693**	−0.1013
BAE200_3	BAE200	−0.0648	−0.2375	−0.3671	**0.6693**	0.0000

Note. The original turquoise (4,015 genes) and blue (1,813 genes) module definitions were retained throughout the sensitivity analysis. Each of the 15 RNA-seq samples was omitted sequentially, and module eigengenes were recalculated from log_2(TPM + 1) values for the remaining 14 samples. Each treatment trait was coded using a one-vs-rest binary scheme, and Spearman correlations were calculated between each module eigengene and the five treatment traits. BAE20 remained the unique strongest positively associated trait for both modules in all 15 iterations, with no ties or replacement by another treatment trait. This analysis evaluates the sensitivity of the fixed module–trait ranking to individual samples and does not assess module membership, network-edge or hub-ranking stability, nor does it constitute independent validation.

Supplementary Table S10 | Hypothesis families and interpretation

Analysis	Multiplicity family	Procedure	Interpretation / limit
Heart rate; speed; each staining/morphometric endpoint	4 exposure-vs-control contrasts separately per endpoint	Holm	Comparisons were adjusted within each endpoint; no adjustment was applied across different endpoints.
Each redox analyte	4 exposure-vs-control contrasts per analyte	Dunnett	Comparisons were adjusted within each analyte; no overall adjustment was applied across the five redox measures.
Each RT-qPCR gene	4 exposure-vs-control contrasts per gene	Two-sided Welch's t-tests; Holm adjustment within each gene	Comparisons were adjusted within each gene; no overall adjustment was applied across different genes.
Formal RNA-seq DEGs	Tested genes within one concentration-vs-control contrast	BH adjusted $P < 0.05$; absolute FC ≥1.5	FDR was controlled separately for each exposure-vs-control comparison.
IPA	Canonical pathways in each existing export	Within-export BH used for ranking	Exploratory pathway analysis based on nominal-*P* gene lists.
WGCNA / STRING	Module–trait tests / data-dependent candidate selection	Nominal P; degree ranking	Used to explore treatment-associated modules and prioritize candidate genes.
Selected gene sets / RANKL:OPG	Descriptive displays	No confirmatory testing claimed	Post hoc descriptive analysis.
Hatching	4 treatment-versus-control contrasts from the longitudinal model	Longitudinal binomial GEE; Holm-adjusted treatment-versus-control comparisons	Repeated observations from the same embryo were accounted for in the model.
Movement-state composition	4 joint treatment-versus-control comparisons	ILR-transformed compositions analyzed using a Euclidean pseudo-F test with 20,000 permutations; Holm adjustment for the four treatment-versus-control comparisons	The three movement components were analyzed together rather than as separate outcomes.

No single endpoint was designated in advance as the primary confirmatory endpoint. Multiplicity was controlled within the comparison families defined above.

Supplementary Table S11 | RANKL/OPG mRNA ratios

TBBPA-BAE (µg/L)	Relative tnfsf11 (RANKL)	Relative tnfrsf11b (OPG)	RANKL/OPG ratio	Ratio relative to control	$\log_2$ ratio shift
Control	1.00 ± 0.02	1.00 ± 0.04	1.00 ± 0.03	1.00	0.00
0.2	2.36 ± 0.03	1.91 ± 0.12	1.25 ± 0.10	1.24	0.31
2	1.56 ± 0.10	1.72 ± 0.08	0.90 ± 0.03	0.90	−0.15
20	2.68 ± 0.09	4.37 ± 0.42	0.62 ± 0.04	0.62	−0.70
200	1.82 ± 0.12	1.52 ± 0.08	1.21 ± 0.14	1.21	0.26

Note. Values are mean ± s.e.m. from three independent biological replicates (20 pooled larvae per replicate) at 14 dpf. The RANKL/OPG ratio was calculated within each replicate as relative tnfsf11 expression divided by relative tnfrsf11b expression; the table reports the mean of the paired replicate ratios. This mRNA-based index is descriptive and does not by itself establish pathway activity or bone resorption.

Supplementary Figure S1 | Additional craniofacial morphometric results and representative Alcian blue measurement landmarks

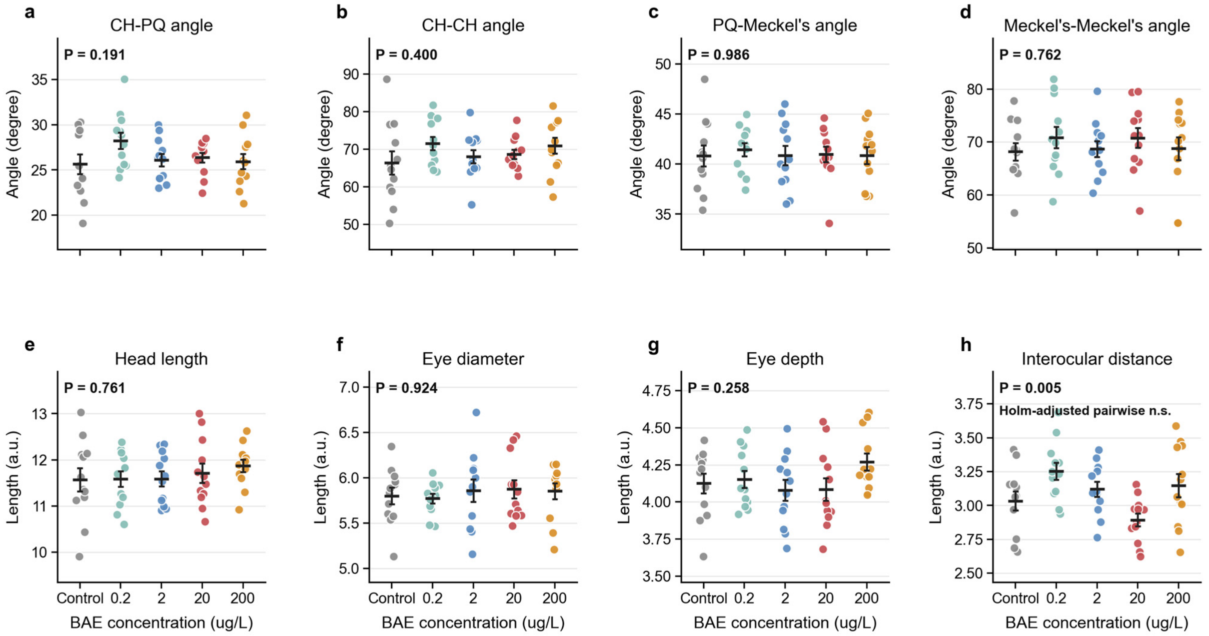

Supplementary Figure S1 | Additional craniofacial morphometric results and representative Alcian blue measurement landmarks. (A–D) CH–PQ angle, CH–CH angle, PQ–Meckel's angle and Meckel's–Meckel's angle. (E–H) Head length, eye diameter, eye depth and interocular distance in 5-dpf larvae exposed to vehicle control or nominal 0.2, 2, 20 or 200 µg/L TBBPA-BAE. Points represent individually exposed larvae, each originating from a separate exposure well; horizontal lines and error bars show mean ± s.e.m. (*n* = 12 independent exposure wells per treatment, with one larva per well). Overall P values were obtained using one-way label-permutation tests: 0.191, 0.400, 0.986, 0.762, 0.761, 0.924, 0.258 and 0.005 for panels A–H, respectively. Each exposure group was compared with the control using two-sided label-permutation tests with Holm correction; no pairwise comparison reached Holm-adjusted $P < 0.05$. Panels A–H are study data. Panel I, on the following page, is a photographed representative Alcian blue-stained control larva in ventral view with direct labels for the three cartilage elements used in the angular measurements (Meckel's cartilage, palatoquadrate and ceratohyal). It clarifies the linear endpoints and angular endpoint definitions without adding a quantitative comparison or reconstructing experimental ROI geometry.

Supplementary Figure S1 (continued) | Representative Alcian blue cartilage image and measurement landmarks

I Representative 5-dpf control, ventral view (Alcian blue)

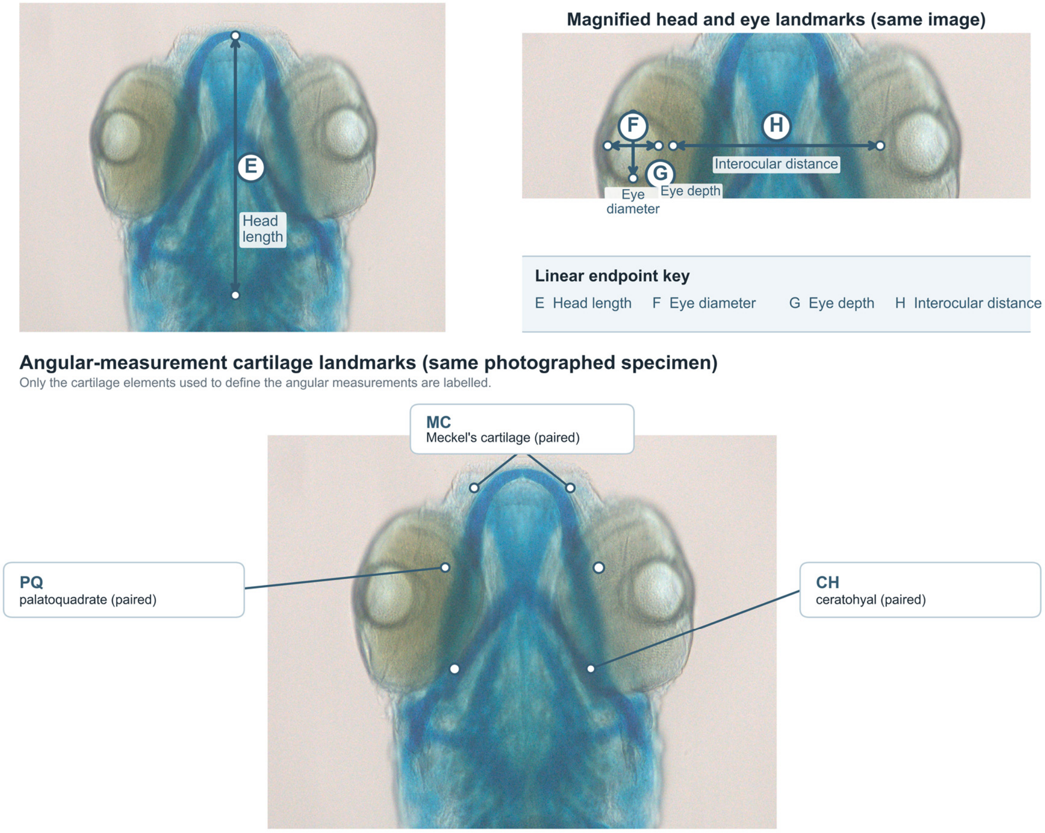

Angle pairs: A, CH-PQ | B, CH-CH | C, PQ-Meckel's cartilage | D, Meckel's-Meckel's cartilage

Anatomical labels identify measured landmarks only; they are not reconstructions of the unretained ImageJ angular ROIs.

Supplementary Figure S1 (continued) | Representative Alcian blue cartilage image and measurement landmarks. (I) A photographed representative 5-dpf control larva after Alcian blue staining in ventral view. The upper panels show the linear endpoints (head length, eye diameter, eye depth and interocular distance). The lower map labels only the cartilage elements used to define the angular metrics: Meckel’s cartilage (MC), palatoquadrate (PQ) and ceratohyal (CH). Solid leaders mark the reference locations, and counterpart dots show the paired elements. Labels are anatomical location guides, not reconstructions of original ImageJ angular ROIs. No intensity, contrast or colour adjustment was applied; only crops and annotation overlays were added.

Supplementary Figure S2 | RT-qPCR primer standard curves

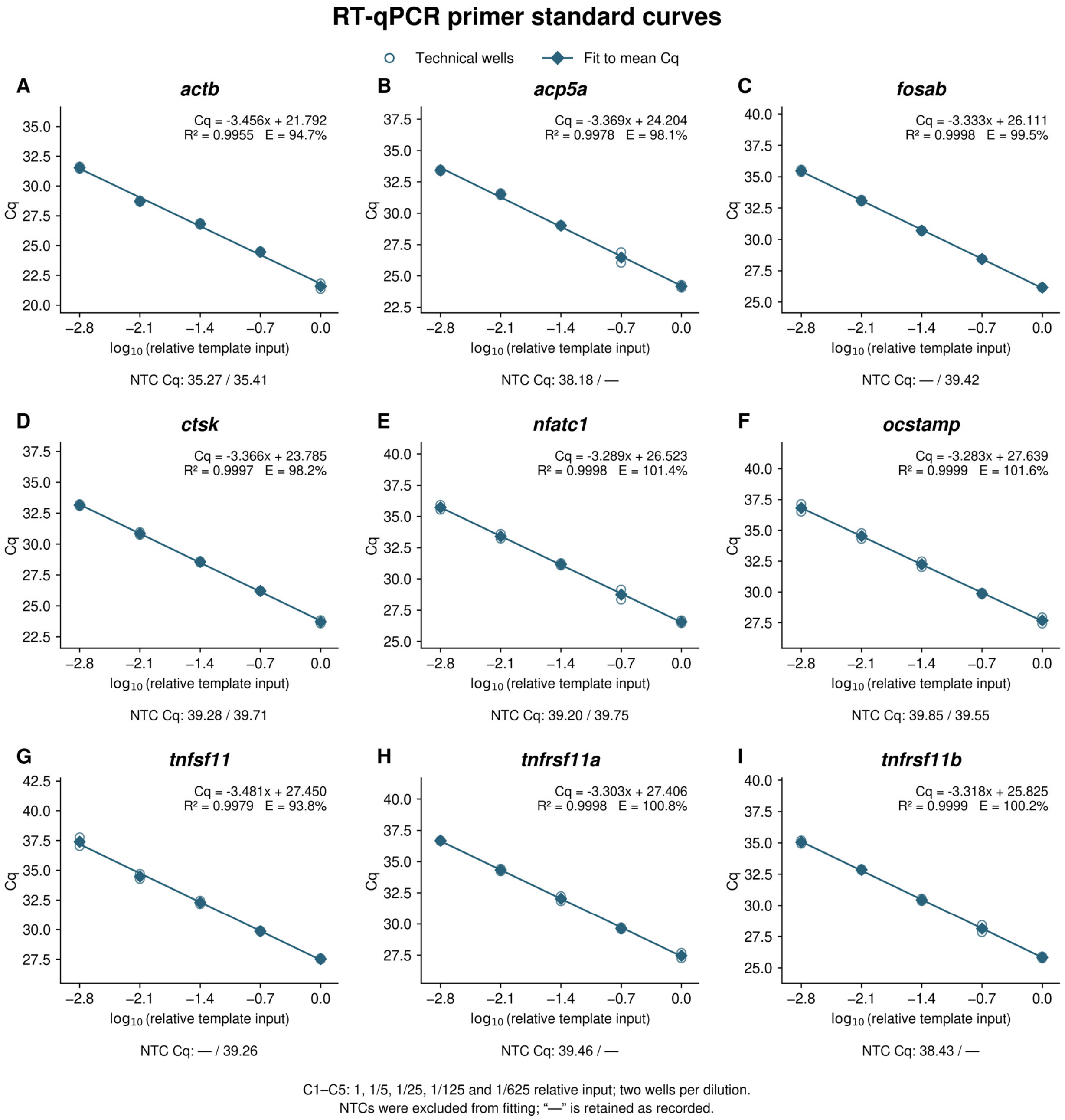

Supplementary Figure S2. RT-qPCR primer standard curves and amplification efficiencies. Five-fold serial dilutions of cDNA were evaluated at five relative template inputs (C1–C5: 1, 1/5, 1/25, 1/125 and 1/625), with two technical wells per dilution. Open circles show individual Cq values, and diamonds show the mean at each dilution. Lines represent ordinary least-squares regression of mean Cq against $\log_{10}$(relative template input). Equations, coefficients of determination (R^2, based on the five means), and amplification efficiencies are shown for each primer pair. Efficiency was calculated as E (%) = [10^(−1/slope) − 1] × 100. NTC Cq values are reported separately and excluded from fitting; dashes are retained as recorded. These standard curves alone do not establish amplification specificity or the lower limit of quantification.

Supplementary Figure S3 | Representative RT-qPCR melting-peak profiles

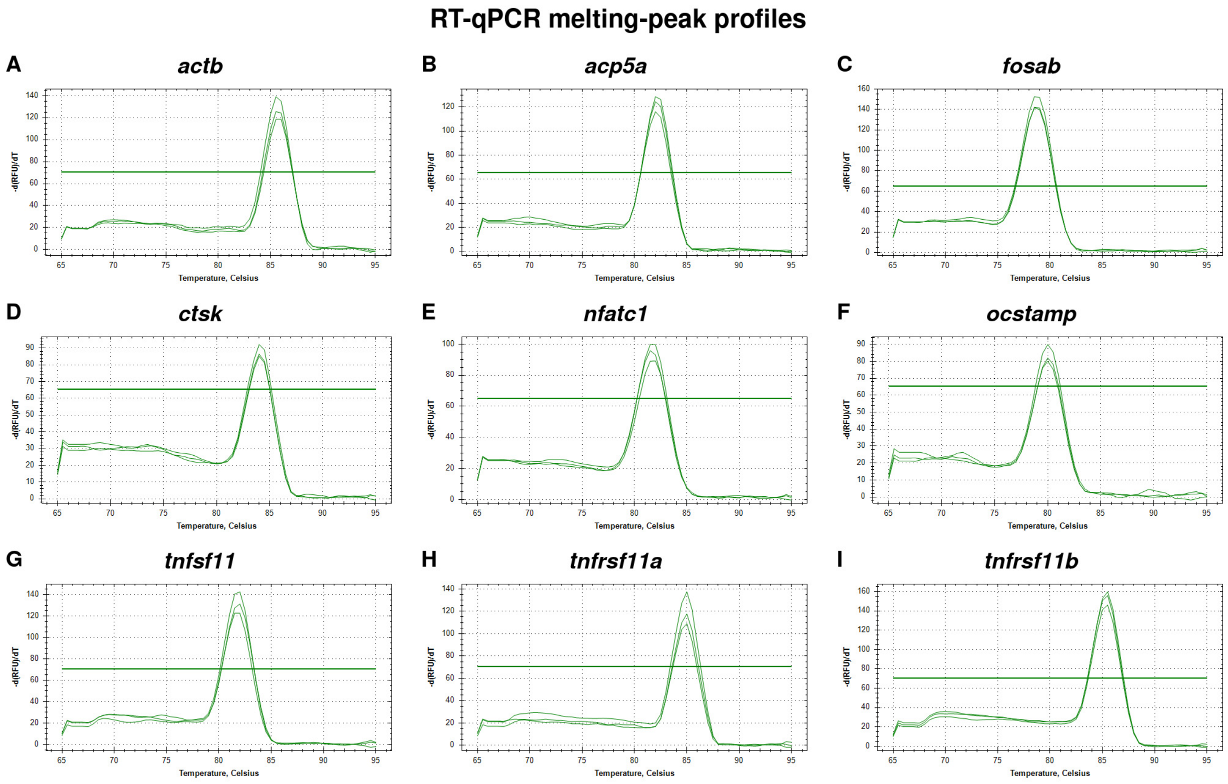

Supplementary Figure S3. Representative RT-qPCR melting-peak profiles. (A) actb; (B) acp5a; (C) fosab; (D) ctsk; (E) nfatc1; (F) ocstamp; (G) tnfsf11 (RANKL); (H) tnfrsf11a (RANK); and (I) tnfrsf11b (OPG). Panel labels and gene order correspond to Supplementary Figure S2. Each panel shows three selected reaction-well traces from the instrument output, plotted as the negative first derivative of fluorescence with respect to temperature (−dRFU/dT). The displayed reactions show a single dominant melting peak. Original curves, axis scales and horizontal software reference lines are retained.

Supplementary Figure S4. Selected KEGG GSEA pathways across TBBPA-BAE concentrations

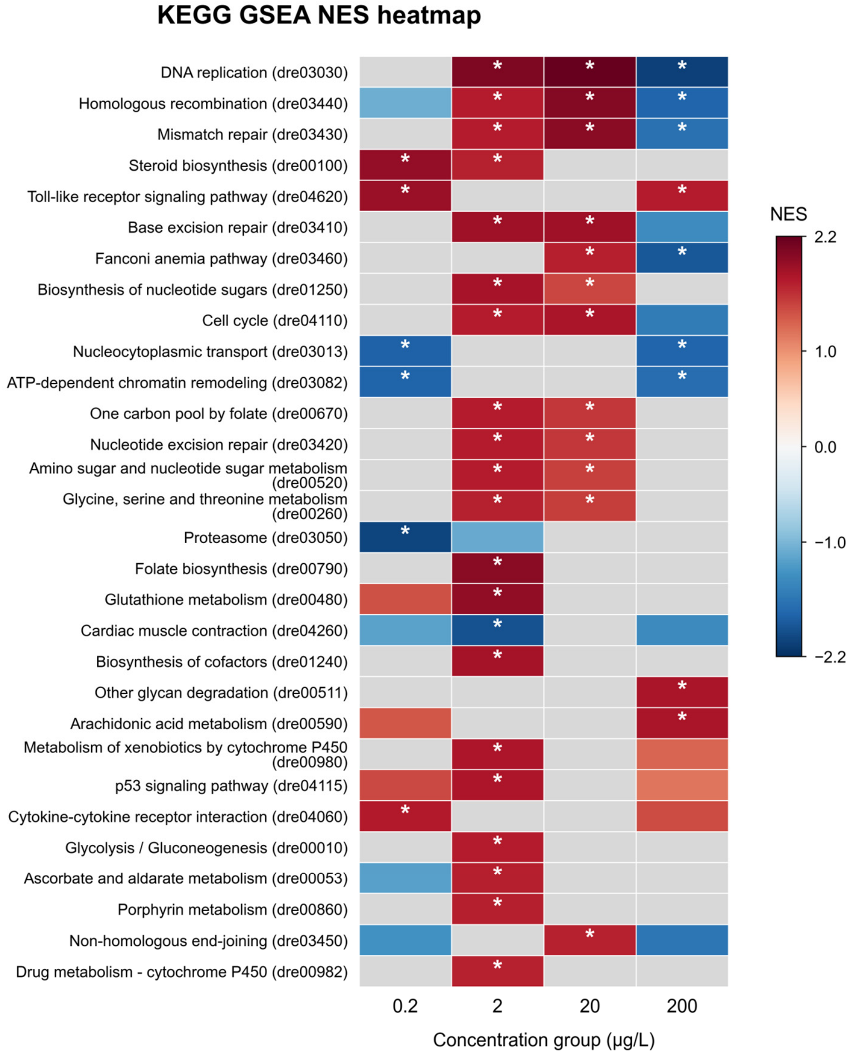

Rows: 30 selected pathways with Padj < 0.05 in at least one concentration, prioritized by the number of significant concentrations and |NES|. * marks Padj < 0.05. Gray cells indicate pathways absent from the corresponding input table.

Supplementary Figure S4. Selected KEGG pathways across TBBPA-BAE concentrations identified by ranked-set gene set enrichment analysis (GSEA). KEGG GSEA was performed separately for each exposure-versus-control comparison using the full ranked gene list. The present heatmap is a secondary visualization based on the existing concentration-specific GSEA output tables and does not recalculate enrichment statistics. Pathways with adjusted P < 0.05 in at least one exposure group were prioritized by the number of significant concentrations, maximum absolute normalized enrichment score (NES), minimum adjusted P value, and pathway representation across concentrations; the top 30 pathways are shown. Cell colors represent NES, with positive NES (red) indicating enrichment toward the exposure group and negative NES (blue) indicating enrichment toward the control group or relative reduction in the exposure group. Asterisks indicate adjusted P < 0.05. Gray cells indicate that the pathway was not present in the corresponding input GSEA table and should not be interpreted as absence of enrichment.

Supplementary Figure S5 | Craniofacial area and its relationship with mineralization at 14 dpf

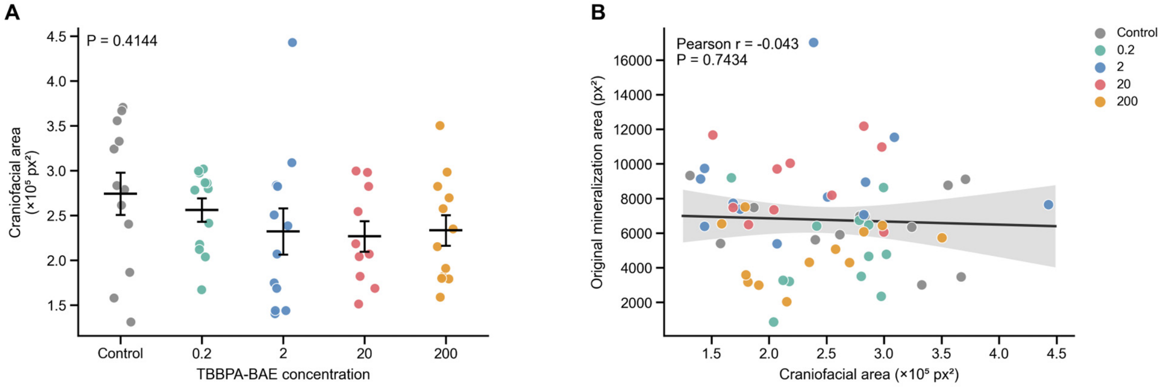

Supplementary Figure S5. Craniofacial size assessment in zebrafish at 14 dpf. (A) Craniofacial area quantified from the same images used for Alizarin red analysis. No significant overall difference was detected among treatment groups (P = 0.4144). (B) Relationship between craniofacial area and mineralized area before craniofacial-size normalization across individual larvae. No significant association was detected (Pearson r = −0.043, permutation P = 0.7434). Points represent individual larvae.

Supplementary Dataset S1 | Transcriptomic and network-analysis results

Supplementary Dataset S1 contains a README and the following supporting records:

Formal DEG lists and concentration-specific DEG summaries were generated using a Benjamini–Hochberg-adjusted P value < 0.05 and an absolute fold change of at least 1.5 ($|\log2FC| \geq 0.585$). Separately, exploratory IPA input gene lists were generated using nominal DESeq2 P < 0.05 and an absolute fold change of at least 1.5 ($|\log2FC| \geq 0.585$). The broader nominal-P lists were used only for exploratory pathway analysis and were not classified as formal DEGs.

The recurrent IPA pathways displayed in Fig. 5C were ranked using the minimum within-export Benjamini–Hochberg FDR across concentrations, followed by the maximum $-\log10(P)$, and the 15 highest-ranked pathways were retained.

- the complete concentration-specific candidate table used to define the exploratory IPA input lists, comprising genes with nominal DESeq2 P < 0.05 and an absolute fold change of at least 1.5;
- formal FDR-and-fold-change DEG lists;
- concentration-specific formal DEG summary;
- concentration-specific KEGG GSEA output tables for BAE0.2, BAE2, BAE20 and BAE200 versus control, including ES, NES, P value, adjusted P value, rank at maximum enrichment and leading-edge information;
- exploratory IPA input gene lists;
- complete IPA pathway outputs;
- WGCNA and STRING supporting results;
- underlying TPM values for the genes displayed in Figs. 5D – E and 6D.
